# Combining multiple spatial statistics enhances the description of immune cell localisation within tumours

**DOI:** 10.1038/s41598-020-75180-9

**Published:** 2020-10-29

**Authors:** Joshua A. Bull, Philip S. Macklin, Tom Quaiser, Franziska Braun, Sarah L. Waters, Chris W. Pugh, Helen M. Byrne

**Affiliations:** 1 Wolfson Centre for Mathematical Biology, Mathematical Institute, University of Oxford, Oxford, OX2 6GG UK; 2 Nuffield Department of Medicine, NDM Research Building, University of Oxford, Oxford, OX3 7FZ UK; 3 Roche Pharma Research and Early Development, pRED Informatics, Roche Innovation Center Munich, Munich, Germany; 4 Oxford Centre for Industrial and Applied Mathematics, Mathematical Institute, University of Oxford, Oxford, OX2 6GG UK

**Keywords:** Cancer imaging, Tumour immunology, Applied mathematics, Statistics, Statistical methods, Computational biology and bioinformatics, Data processing, Systems biology, Imaging the immune system, Tumour immunology

## Abstract

Digital pathology enables computational analysis algorithms to be applied at scale to histological images. An example is the identification of immune cells within solid tumours. Image analysis algorithms can extract precise cell locations from immunohistochemistry slides, but the resulting spatial coordinates, or point patterns, can be difficult to interpret. Since localisation of immune cells within tumours may reflect their functional status and correlates with patient prognosis, novel descriptors of their spatial distributions are of biological and clinical interest. A range of spatial statistics have been used to analyse such point patterns but, individually, these approaches only partially describe complex immune cell distributions. In this study, we apply three spatial statistics to locations of CD68+ macrophages within human head and neck tumours, and show that images grouped semi-quantitatively by a pathologist share similar statistics. We generate a synthetic dataset which emulates human samples and use it to demonstrate that combining multiple spatial statistics with a maximum likelihood approach better predicts human classifications than any single statistic. We can also estimate the error associated with our classifications. Importantly, this methodology is adaptable and can be extended to other histological investigations or applied to point patterns outside of histology.

## Introduction

The importance of the immune response to tumour biology and therapy is increasingly recognised and improved understanding is required. Immune responses are multifaceted, with different functions being mediated by individual leukocyte sub-types. These leukocyte sub-types (and some aspects of their function) are defined by the expression of unique proteins. The location within tumour material of these proteins, and thus the cells expressing them, can be identified by immunohistochemistry^1^. Since approximately 50% of a tumour is composed of host-derived stroma, knowledge of which immune cells infiltrate the regions occupied by malignant cells is key to understanding their function in influencing tumour biology^2–4^. In this study we use as an exemplar the distribution of cells expressing CD68, a protein expressed by macrophages.

In current clinical practice assessment of leukocyte infiltration into tumours is undertaken by highly trained histopathologists using semi-quantitative approaches. Detailed analysis is limited by the effort required to make the measurements, concerns over inter-observer variability and uncertainty about how to handle the manifest biological variability present in tumour material. Despite these issues, current estimates of leukocyte infiltration provide clinically useful information that is both helpful in predicting prognosis and guiding treatment decisions^5–7^. It is likely that deeper analysis would reveal currently untapped information, providing an impetus to develop methods that are more quantitative and can be automated.

Digital pathology provides new opportunities to improve and automate tasks^8,9^ such as cell counting^10,11^, identifying structures within tumours^12,13^, and classification and scoring of routine clinical parameters^14–16^. However, it introduces new challenges related to the size of imaging datasets and variability of staining from slide to slide^17^. While existing open source^18–21^ and commercial software alternatives can be used to extract (*x*, *y*)-coordinates of cell centres, we use a pipeline which allows relatively robust and facile identification of cell locations (described in the Supplementary Information, Sects. A, B). The availability of (*x*, *y*)-coordinates for each cell of a given type allows their distribution to be described using spatial statistics. Individual spatial statistics provide quantitative mathematical descriptions which have varying degrees of correlation with histopathological classifications^22–30^. Importantly, we show that by combining spatial statistical descriptions of this data we can automatically derive information which is comparable to that currently reported by a pathologist and more accurate than that obtained using a single spatial statistic.

Differences in tissue architecture, cellular morphology and staining intensity permit manual discrimination between tumour cell nests and adjacent stroma in histology images. However, automated tissue compartmentalisation is not straightforward. Whilst several hand-crafted machine learning approaches have been developed^31–33^, this task may be best performed through deep learning^34–36^, a technique that is not yet routinely available. Therefore, our statistical method considers only the (*x*, *y*)-coordinates of the point patterns formed by cell centres and does not depend upon the identification of the tumour/stroma boundary. Furthermore, by using only (*x*, *y*)-coordinates of cell centres there is also no requirement for multiplex immunohistochemistry or depletion of tumour tissue by labelling adjacent histological sections for other markers, although the methodology described here can be easily adapted to incorporate additional information generated by such approaches.

The approaches we describe provide a route to explore extra information about leukocyte distribution that is not specifically captured by current pathology classifications. Examples could include heterogeneity of infiltration (explored in this report), cellular co-localisation and position relative to tumour micro-environmental measures. By providing precise quantitation, this methodology will allow the underlying biological significance and mechanisms to be explored and hopefully be of both scientific and clinical utility.

## Methodology

### Datasets

We consider two types of point patterns; cell centroids extracted from human head and neck cancer IHC slides, and computer generated synthetic infiltration patterns. Other tumour types are discussed in the Supplementary Information (Sect. F).

#### Head and neck cancer: regions of interest

A cohort of 16 resected human head and neck tumours was stained to show macrophage locations (CD68+). 4  μm sections were cut from formalin-fixed paraffin embedded tissue blocks of 16 cases of human head and neck squamous cell carcinoma. The sections underwent immunohistochemistry staining on a Leica BOND-MAX automated staining machine (Leica Biosystems). Briefly, sections were deparaffinized, underwent epitope retrieval and endogenous peroxidase activity was blocked with 3% hydrogen peroxide (5 min). Subsequently, sections were incubated with the primary antibody (30 min) followed by post-primary and polymer reagents (8 min each). Next, 3,3′-Diaminobenzidine (DAB) chromogen was applied (10 min) and slides were counterstained with haematoxylin (all reagents included within the BOND Polymer Refine Detection kit, Leica Biosystems, catalogue no. DS9800). The following primary antibody was used during staining: CD68 (mouse monoclonal, clone PG-M1), Agilent Technologies, UK (catalogue no. M087601-2), 1:200 concentration. The positive control sample comprised a section of human tonsil tissue. Stained slides were scanned at ×200 magnification using the NanoZoomer S210 digital slide scanner (Hamamatsu). Whole slide images were reviewed by a pathologist (PSM) who annotated tumour regions and any artefactual changes. Non-overlapping 1.5 mm × 1.5 mm regions of interest (ROIs) were then randomly sampled from within the tumour region on each slide until the region was saturated. This resulted in 549 1.5 mm × 1.5 mm ROIs from across the cohort. Centroids of CD68+ cells were extracted from each ROI using a custom image analysis pipeline (see Supplementary Information, Sects. A, B).

As previously reported^37^, patients were approached and informed consent obtained for use of their tissue. Access to these tissue samples for this study was approved under Oxford Radcliffe Biobank (ORB) research tissue bank ethics, reference 09/H0606/5+5 (approved by the National Research Ethics Service [NRES] Committee South Central—Oxford C). All experimental protocols were approved prospectively by the ORB committee and subsequently conducted in accordance with its conditions and those of NRES.

#### Synthetic datasets

The process for generating synthetic data is described in detail in the Supplementary Information, Sect. D. Briefly, we use Gaussian random fields to divide each 1.5 mm × 1.5 mm square into two compartments representing stroma and tumour cell nests. The relative widths of tumour cell nests and stromal structures within the artificial tumour geometries are defined by a characteristic tumour cell nest length scale *l*. An artificial geometry is accepted only if at least 25% of its area is covered by each compartment (tumour or stroma). The number of points in each compartment of the synthetic ROI is controlled to maintain a specified overall cell density *d* cells per mm^2^ (the impact of varying *l* and *d* on point patterns is explored in Sect. G of the Supplementary Information). The relative density of simulated immune cells in the tumour cell nest ($d_t$) and the stroma ($d_s$), is controlled by a parameter $\rho {} = \frac{d_t}{d_s}$ which further restricts point placement: low values of $\rho$ generate patterns for which the majority of points are excluded from the tumour cell nest, while values close to 1 generate patterns for which points are uniformly distributed across both regions. Candidate points are sampled randomly from the ROI, and accepted if they do not invalidate these criteria. Points are also rejected if they fall within an exclusion radius of 20 μM of any other point, representing approximately one cell diameter.

The macrophage density across the cohort of head and neck ROIs is approximately normally distributed with mean 333 cells per mm^2^ and standard deviation 170 cells per mm^2^. We therefore sample values of *d* from this distribution when generating synthetic data for testing and validation. We reject distributions having $d < 150$ cells per mm^2^ to ensure reasonable cell densities when calculating spatial statistics (lower densities are discussed in the Supplementary Information, Sect. E). We construct a training dataset by generating at least 1200 point clouds for values of $\rho$ in the interval [0, 0.5] (increments = 0.02), and *l* sampled uniformly from the interval [0.1 mm; 0.75 mm]. A separate validation dataset, comprising a further 3680 point clouds, was generated using the same method but with $\rho$ sampled uniformly and at random from the interval [0, 0.5].

### Spatial statistics

**Figure 1:**
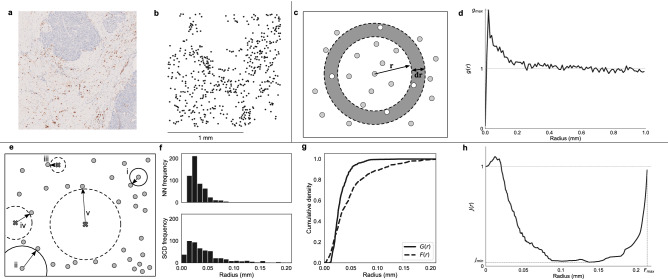
(**a**) 1.5 mm × 1.5 mm ROI from the head and neck dataset in which the macrophages (CD68+) are stained brown and the tumour nests pale blue. (**b**) Point cloud of macrophage locations extracted from the image in (**a**). (**c**) Schematic showing calculation of the PCF, *g*(*r*). An annulus of width *dr* and radius *r* is placed around each point, and the density of points which fall within the annulus is calculated. This density is normalised by the expected density of points within the annulus under complete spatial randomness (CSR) to give *g*(*r*). (**d**) The PCF, *g*(*r*), for the point cloud in (**b**). (**e**) Schematic illustrating distances used to calculate the nearest neighbour (NN) distribution and spherical contact distribution (SCD); circles = cell centres; crosses = randomly chosen locations. The NN distribution describes the smallest distance between points in the point pattern (circle to circle: i, ii, radius shown as solid line) while the SCD describes distances from randomly chosen locations to points (cross to circle, iii, iv, v, radius shown as dashed line). (**f**) Nearest neighbour distribution (NN) and SCD for the point pattern in (**b**). (**g**) Cumulative distribution functions (CDFs) associated with the histograms in (**f**). The function *G*(*r*) is the NN distribution CDF, and *F*(*r*) is the CDF of the SCD. (**h**) The $J$-function for the point cloud in (**b**), calculated from the CDFs in (**g**). $F_\text {max}$ approximates the size of the largest void in the point pattern; it is the largest radius observed in the SCD [see location v in panel (**e**), where the dashed radius is $F_\text {max}$ for these points]. The $J$-function describes differences in the distribution of points from complete spatial randomness. For a given *r*, smaller values of *J*(*r*) suggest clustering, in that more cells will be found in a circle of radius *r* centred at a cell than in a randomly placed circle. $J_\text {min}$ describes how densely the cells are clustered across all possible length scales.

We consider three spatial statistics: the pair correlation function (PCF), the spherical contact distribution (SCD) and the $J$-function. Figure 1 shows typical results when these statistics are applied to a ROI from the head and neck cancer dataset.

#### Pair correlation function, *g*(*r*)

The PCF (or radial distribution function)^38–40^ compares the average density of points against complete spatial randomness (CSR) across length scales. It has previously been used to estimate the length scales of emergent patterns in point clouds representing the locations of distinct cell types^41^. To generate the PCF, an annulus of width *dr* and radius *r* is placed around each point (Fig. 1c). The number of points within each annulus is calculated and normalised with respect to the expected number of points that would fall inside the annulus under CSR. This calculation is repeated for each point, and then the average value is recorded as a measure of whether points are more or less frequently observed at distance *r* from a point than expected under CSR. This is repeated for a range of *r* to generate the PCF, *g*(*r*). Figure 1d shows the PCF obtained from the point pattern in Fig. 1b. Further details, including handling of boundary conditions and edge effects, can be found in the Supplementary information, Sect. C. The PCF identifies clustering at distance *r*: $g(r) > 1$ indicates that pairs of points separated by distance *r* occur frequently, while $g(r) < 1$ indicates that pairs of points are less likely to be separated by distance *r* than under CSR. In the case of CSR generated through a Poisson process, $g(r) \equiv 1$^40^. In the context of CD68+ macrophages, $g(r) < 1$ indicates that macrophages are rarely observed separated by radius *r* (occurring only at very small distances, $r \approx 0$ in Fig. 1d. We interpret this as a minimum distance that cell centres must be separated by, corresponding approximately to the radius of a macrophage). $g(r) > 1$ indicates that macrophages are likely to be separated by distance *r*. This occurs for $r < 0.2\text { mm}{}$ in Fig. 1d, showing short-range clustering of macrophages.

#### Spherical contact distribution

The SCD (or empty-space function)^42,43^ is closely related to the nearest-neighbour distribution. Figure 1e–g show how the two functions are constructed. The nearest-neighbour distribution [shown in Fig. 1f for the point pattern in Fig. 1b] is obtained by measuring the distance from each point to its nearest neighbour. The SCD is calculated in a similar way, but reference points are selected randomly from the ROI rather than from the point cloud. For each randomly chosen point, the distance to its nearest neighbour in the point cloud is added to the distribution. Each observation of radius *r* in the SCD corresponds to a circle of radius *r* containing no points. Examples of these circular voids can be seen in Fig. 1e, surrounding points iii, iv and v. In the context of CD68+ macrophages, observations at radius *r* in the SCD therefore indicate the absence of macrophages in a circle of radius *r* somewhere in the region.

#### $J$-function, *J*(*r*)

The $J$-function is a non-parametric test for identifying clustering and dispersion in point patterns^42^. The $J$-function compares the cumulative density functions (CDFs) of the SCD, *F*(*r*), and the nearest-neighbour distribution function, *G*(*r*), [shown in Fig. 1g for the point pattern in Fig. 1b], where

1 $$\begin{aligned} F(r) = \frac{\text {Number of randomly chosen reference points within distance $r$ of a target point}}{\text {Number of randomly chosen reference points}}, \end{aligned}$$

and

2 $$\begin{aligned} G(r) = \frac{\text {Number of points within distance $r$ of another point}}{\text {Number of points}}, \end{aligned}$$

so that $0 \le F(r) \le 1$ and $0 \le G(r) \le 1$. When calculating *F*(*r*) we choose the same number of reference points as there are points in the pattern to ensure that the denominators of Eqs. (1) and (2) are equal. The *J*-function is defined as:

3 $$\begin{aligned} J(r) = \frac{1 - G(r)}{1 - F(r)}. \end{aligned}$$

Under CSR $G(r) \approx F(r)$, and hence $J(r) \approx 1$. If $J(r) > 1$ then more points are found in a circle of radius *r* placed randomly in the domain than in a circle centred at one of the points, indicating dispersal of points within the point cloud. If $J(r) < 1$ then a disc of radius *r* centred on a point contains more points than a randomly placed disc, and we conclude that the points are clustered^42^. Figure 1h shows *J*(*r*) for the point pattern in Fig. 1b.

In the context of CD68+ macrophages, $J(r) > 1$ indicates dispersal of macrophages; this is observed at small values of *r* in Fig. 1h. As with the PCF in Fig. 1d, this indicates that macrophage cell centres are rarely separated by distances of less than 0.02 mm (i.e., the approximate radius of a macrophage.) $J(r) < 1$ indicates clustering of macrophages, which we see at length scales up to approximately 0.2 mm, again correlating with the PCF.

### Summary statistics

We record three features of the spatial statistics: the peak of the PCF, $g_\text {max}$; the value *r* at which $F(r) = 1$, $F_\text {max}$; and, the minimum value of the $J$-function, $J_\text {min}$. These features have the following interpretations:

- $g_\text {max}{} := \max (g)$. *g*(*r*) describes the expected density of points at distance *r* from another point, compared to CSR. Hence $g_\text {max}$ describes the maximum intensity of point clustering over all radii *r*.
- $F_\text {max}{} := \min (r)$ such that $F(r) = 1$. $F_\text {max}$ approximates the radius of the largest circular void in the point distribution. As macrophages start to infiltrate into these ‘immune deserts’ in greater numbers, the voids in the point pattern should reduce in size, and $F_\text {max}$ will also decrease.
- $J_\text {min}{} := \min (J)$. As $J(0) = 1$ and $J \ge 0$, we have $0 \le J_\text {min}\le 1$. Smaller values of $J_\text {min}$ indicate that a larger number of macrophages are closer to other macrophages than to randomly selected points (i.e., denser cell clustering).

### Maximum likelihood estimation

We conduct maximum likelihood estimation (MLE) based on observations of $g_\text {max}$, $F_\text {max}$ and $J_\text {min}$ in order to predict $\rho$ for a given synthetic point pattern. While the likelihood can be estimated directly from the empirical distributions, we instead approximate distributions of $g_\text {max}$, $F_\text {max}$ and $J_\text {min}$ using a normal distribution for each $\rho$. The mean and standard deviation (SD) of these distributions is well approximated using exponential functions of $\rho$ (shown in Fig. 3b–d). This approximation ensures that the maximum likelihood predictions vary monotonically with $g_\text {max}$, $F_\text {max}$ or $J_\text {min}$. This approximation is discussed in the Supplementary Information, Sect. H. As we assume that for each $\rho$ the distributions of $g_\text {max}$, $F_\text {max}$ and $J_\text {min}$ are normal, the log likelihood, $\ln (L)$, can be calculated directly as:

4 $$\begin{aligned} \ln {L} = -0.5 \left( \ln {(2 \pi \sigma ^2) + \frac{(x - \mu )^2}{\sigma ^2}} \right) \end{aligned}$$

when only one spatial statistic is used, where $\mu$ and $\sigma$ are the mean and SD of the distribution of the statistic, and *x* is the value of the statistic. When observations from *k* different statistics are combined, Eq. (4) generalises to give:

5 $$\begin{aligned} \ln {L} = -0.5 \left( \ln {|\Sigma |} + (\mathbf {x} - \mathbf {\mu })^T \Sigma ^{-1} (\mathbf {x} - \mathbf {\mu }) + k\ln {(2 \pi )} \right) \end{aligned}$$

where $\mathbf {x}$ is the vector of observations, $\mathbf {\mu }$ is the vector of the means of the distributions and $\Sigma$ is the covariance matrix. While $\mathbf {\mu }$ and $\mathbf {\sigma }$ are functions of $\rho$ (Supplementary Information, Sect. H), $\Sigma$ must be estimated from the empirical data.

For each $\rho$ we generate a *k*-dimensional grid containing $\ln {L}$ at evenly spaced values of the spatial statistics. These grids are stacked to form a $k+1$ dimensional lookup table. For an observation of *k* spatial statistics, the closest grid points are taken and the profile likelihood is identified from the remaining dimension. The MLE for $\rho$, which we denote $\eta _{}$, is then estimated. A 95% confidence interval around $\eta _{}$ can be found by identifying where the profile log likelihood is above the threshold $\eta _{} -0.5 \ln {(\chi ^2(0.95,1))}$, where $\chi ^2(0.95,1)$ is the 0.95 quantile of the chi-squared distribution with 1 degree of freedom^44,45^.

## Results

### Application of spatial statistics to histological samples

**Figure 2:**
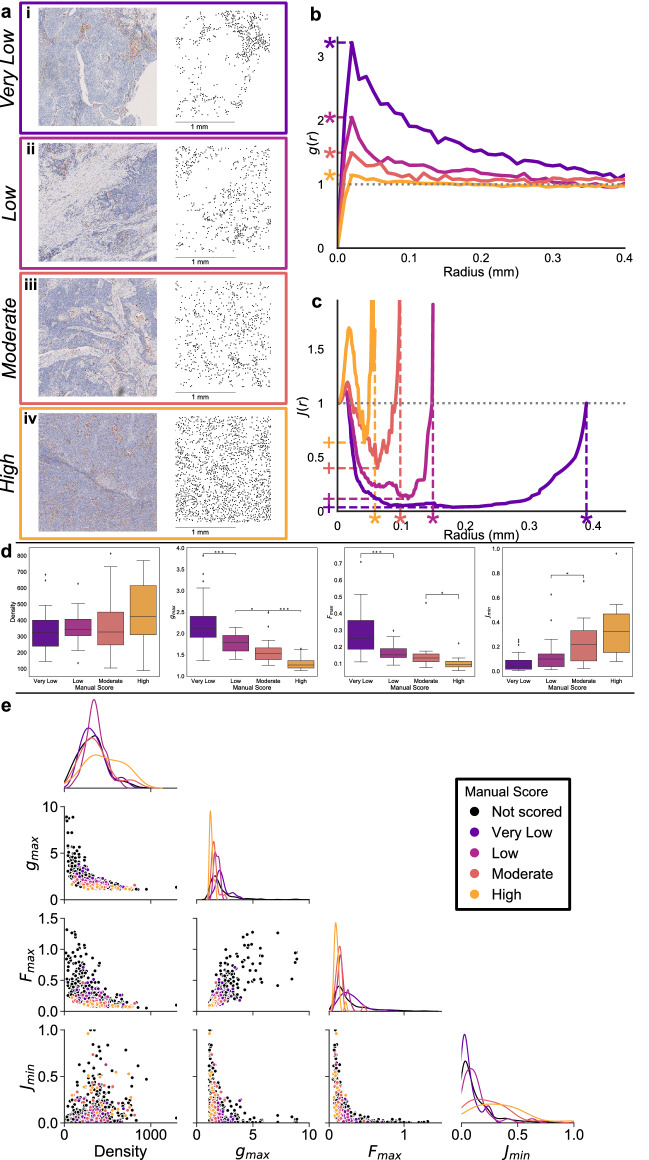
(**a**) 1.5 mm × 1.5 mm ROIs from human head and neck tumours assessed as having (i) very low, (ii) low, (iii) moderate, and (iv) high macrophage infiltration into tumour cell nests, with extracted point clouds. (**b**) PCFs for the point clouds in (**a**). As macrophage infiltration increases, the peak of the PCF ($$g_\text {max}$$, marked ‘*’) tends towards 1. (**c**) $$J$$-functions for the point clouds in (**a**). As macrophage infiltration increases, the minimum of the $$J$$-function ($$J_\text {min}$$, marked ‘+’) tends towards 1, while the domain of definition of the $$J$$-function ($$F_\text {max}$$, marked ‘*’) becomes smaller. (**d**) Distribution of *d*, $$g_\text {max}$$, $$F_\text {max}$$ and $$J_\text {min}$$ according to manual scoring of 89 ROIs. *p* values are the result of *t*-tests on adjacent boxplots. Macrophage density remains consistent across all scoring categories, but $$g_\text {max}$$ and $$F_\text {max}$$ become smaller with increasing infiltration. $$J_\text {min}$$ appears to increase with macrophage infiltration. The differences in $$F_\text {max}$$ between low/moderate infiltration, and in $$J_\text {min}$$ between very low/low and moderate/high infiltration, are not significant for this dataset. $$*: p < 0.05$$, $$**: p < 0.01$$, $$***: p < 0.001$$. **e** Distributions (diagonal) and pairwise combinations (off-diagonal) of *d*, $$g_\text {max}$$, $$F_\text {max}$$ and $$J_\text {min}$$ observed in the head and neck dataset. The 89 labelled ROIs are coloured according to manual scoring, and the full unscored dataset of 549 ROIs is shown in black.

We analysed 549 1.5 mm × 1.5 mm regions of interest (ROIs), taken from 16 human head and neck tumour slides stained to show macrophage locations (CD68+). 100 ROIs were randomly selected for manual evaluation by a pathologist as containing “very low”, “low”, “moderate” or “high” CD68+ macrophage infiltration into tumour cell nests, with 11 subsequently excluded from analysis for containing artefacts such as weak staining or damaged tissue. The pathologist graded CD68+ macrophage infiltration into tumour nests, irrespective of the overall immune cell infiltration within the ROI and without controlling for tumour nest size. CD68+ macrophage point clouds extracted from the remaining 89 ROIs were analysed using the PCF, SCD and $J$-function. Representative ROIs and point clouds are presented in Fig. 2a, while Fig. 2b,c show the PCFs and $J$-functions.

The shapes of the PCF and $J$-function are similar for different ROIs, but properties of the curves change with increasing infiltration (Fig. 2b,c). For each ROI, the PCF is initially low ($g(r)<1$ when $r <20\,\mu m{}$), in keeping with volume-exclusion of cell centroids meaning that macrophages are unlikely to be observed within approximately one cell diameter of one another. This is typically followed by a peak ($g(r)>1$), suggesting short-range clustering.

As *r* increases the PCF decays towards 1 (grey dotted line), showing decreasing colocalisation between macrophages at larger length scales. The maximum value of the PCF ($g_\text {max}$, indicated by ‘*’ and dashed lines) decreases with increased macrophage infiltration and decays more rapidly to its asymptotic value. These trends show that increased infiltration implies a more homogeneous distribution of macrophages across a ROI.

The $J$-function reveals similar behaviour (see Fig. 2c). After an initial peak associated with exclusion of points at $r < 20\,\mu m{}$, the $J$-function drops below 1 to a minimum value $J_\text {min}$ (indicated by ‘+’ and horizontal dashed lines in Fig. 2) and then rises again. $J_\text {min}$ increases with increasing macrophage infiltration. This suggests that CD68+ cells in ROIs with higher infiltration are less densely clustered than in those with low infiltration. The $J$-function is defined only where $F(r) < 1$; we define this radius as $F_\text {max}$ (labelled with ‘*’ and vertical dashed lines in Fig. 2c). As *r* approaches $F_\text {max}$, the $J$-function becomes infinite (see Fig. 2c), suggesting that the distance between a macrophage and its nearest neighbour is never larger than the radius of immune deserts in the IHC ROIs. ROIs with low infiltration contain large areas with no macrophages, and therefore have higher values of $F_\text {max}$, than those with high infiltration.

Figure 2d shows that these trends persist across the 89 manually scored ROIs. The CD68+ macrophage density does not correlate with the manual scores. Both $g_\text {max}$ and $F_\text {max}$ are negatively correlated with manual scores of increased CD68+ cell heterogeneity, whilst $J_\text {min}$ is positively correlated. There are insufficient scored samples to identify statistically significant differences between all of the categories, but these trends suggest that high $g_\text {max}$, high $F_\text {max}$, and low $J_\text {min}$ are characteristic of ROIs with low macrophage infiltration. Figure 2e places these statistics into the context of those from the full, unscored dataset. Similarly scored ROIs exhibit similar combinations of spatial statistics, indicating that, with more labelled data, these descriptors could inform a classifier that predicts the manual scores. In practice, manually scoring ROIs is time consuming. Further, it may be impractical to obtain sufficient labelled data to train a classifier. We explain below how, in such circumstances, synthetic point patterns may be used as a surrogate for developing such a classifier.

### Comparison of synthetic data with head and neck ROIs

**Figure 3:**
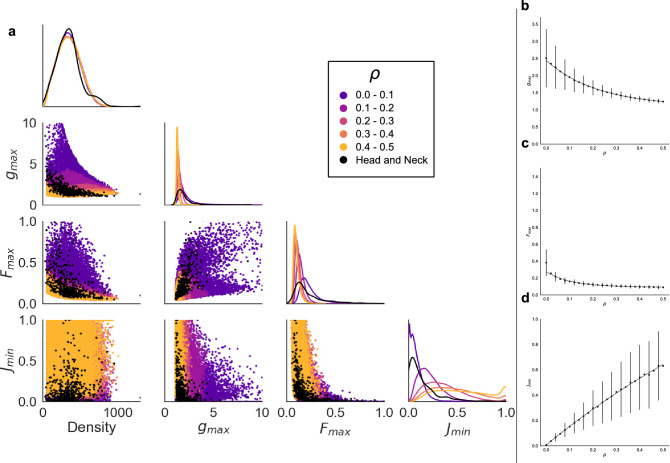
(**a**) Distributions (diagonal) and pairwise correlations (off-diagonal) for *d*, $g_\text {max}$, $F_\text {max}$ and $J_\text {min}$. Statistics drawn from the IHC ROIs are shown in black. Other points represent synthetic ROIs in the training dataset and are coloured according to $\rho$. Distributions of cell density are similar for the head and neck data and all ranges of $\rho$ in synthetic data. The statistics extracted from the head and neck data more closely resemble those of synthetic data with low $\rho$ than high. (**b**–**d**) The effect of increasing the degree of infiltration, $\rho$, on (**b**) $g_\text {max}$, (**c**) $F_\text {max}$ and (**d**) $J_\text {min}$ in the synthetic training dataset. Points are the mean of at least 1200 repetitions of randomly generated point clouds. Vertical lines show the standard deviation, and dashed lines show the best fit of the means to an exponential function: (**b**) $g_\text {max}{} = 1.34 e^{-4.52 \rho {}} + 1.10$, (**c**) $F_\text {max}{} = 0.18 e^{-9.16 \rho {}} + 0.09$, (**d**) $J_\text {min}{} = -2.04 e^{-0.74 \rho {}} + 2.06$.

Figure 3a compares the head and neck ROIs with synthetically generated training data. The distributions of cell density *d*, $g_\text {max}$, $F_\text {max}$ and $J_\text {min}$ are in good agreement with those calculated from the IHC data. The combinations of spatial statistics observed in the head and neck ROIs are a subset of those in the synthetic data, suggesting that the distribution in a given ROI could be approximated by synthetic data with an appropriately chosen $\rho$. We note that the distributions of spatial statistics in the head and neck data are most similar to synthetic data generated with low $\rho$, suggesting that macrophages tend not to be distributed by CSR. Increasing $\rho$ generates point patterns with spatial statistics similar to those from highly infiltrated ROIs (see Fig. 2). Figure 3a therefore suggests that $\rho$ could be used as a label to describe infiltration in synthetic point patterns. Under this assumption, the synthetic training data could be used to identify relationships between spatial statistics and manual scores of IHC ROIs. Combinations of spatial statistics used to infer $\rho$ for synthetic ROIs could also be used to stratify the IHC samples based on macrophage infiltration.

Figure 3b–d shows how varying $\rho$ affects $g_\text {max}$, $F_\text {max}$ and $J_\text {min}$ in the synthetic training dataset. For fixed $\rho$ observations of $g_\text {max}$, $F_\text {max}$ and $J_\text {min}$ are approximately normally distributed, and the mean and standard deviation (SD) of these distributions are well approximated by exponential functions of $\rho$ (dashed lines in Fig. 3b–d; see Supplementary Information, Sect. H). This suggests that the mean and SD of the distributions of $g_\text {max}$, $F_\text {max}$ and $J_\text {min}$ can be estimated for any value of $\rho {} \le 0.5$, including values not present in training data.

### Inference of $\rho$ for synthetic regions

To test whether $\rho$ can be inferred from observations of $g_\text {max}$, $F_\text {max}$ and $J_\text {min}$, we predict $\rho$ for 3680 point patterns in the synthetic testing dataset, where $\rho$ is distributed uniformly at random across the interval [0,0.5]. We use maximum likelihood estimation based on observations of $g_\text {max}$, $F_\text {max}$ and $J_\text {min}$ to infer the value of $\rho$ used to generate each point pattern. We denote the maximum likelihood estimate (MLE) $\eta _{X}$, where $X \subseteq [g,F,J]$ and *g*, *F* and *J* indicate the use of $g_\text {max}$, $F_\text {max}$ and $J_\text {min}$ respectively, giving seven possible combinations of spatial statistics to consider. This notation refers to particular instances of MLE prediction, and we will use $\eta _{}$ with no subscript to refer to MLE predicted from any combination of spatial statistics.

**Figure 4:**
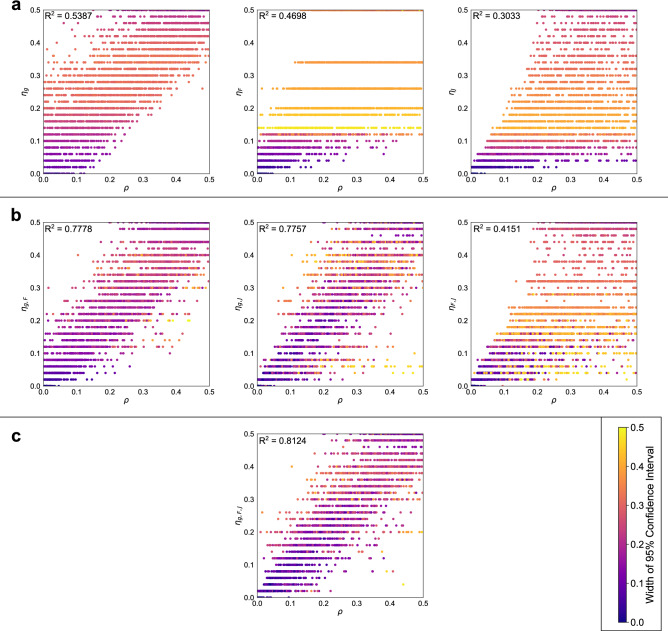
Comparison of $\rho$ against $\eta _{}$, for 3680 synthetic point patterns. Points are coloured according to the width of the 95% confidence interval associated with the prediction. Conducting maximum likelihood estimation (MLE) based on observations of multiple statistics can increase prediction accuracy and tighten confidence intervals. $R^2$ values measure deviation from the line $\rho = \eta _{}$. (**a**) $\rho$ based on observations of one spatial statistic. (**b**) $\rho$ based on observations of two spatial statistics. (**c**) $\rho$ based on observations of three spatial statistics.

Figure 4 shows the MLEs of $\eta _{}$ for different combinations of $g_\text {max}$, $F_\text {max}$ and $J_\text {min}$ for the same point patterns. Each marker corresponds to a different point pattern, and is coloured according to the width of the 95% confidence interval around $\eta _{}$. Predictions in Fig. 4a are based on observations of a single spatial statistic, those in Fig. 4b show predictions of pairwise combinations of the statistics, and Fig. 4c shows predictions made by combining observations of all three statistics. The distance of the predictions to the line $\rho {} = \eta _{X}$ can be evaluated using $R^2$. Predictions made based on individual spatial statistics, $\eta _{g}$, $\eta _{F}$ and $\eta _{J}$, are not strongly predictive [$R^2 = 0.5387, R^2 = 0.4698, R^2 = 0.3033$ respectively, Fig. 4a]. $\eta _{g}$ generally overestimates $\rho$, but is the most accurate of the single spatial statistics. $\eta _{F}$ displays banding caused by rounding of observations of spatial statistics (see Supplementary Information, Sect. H). Estimates of $\eta _{}$ close to 0 have small confidence intervals. The width of confidence intervals is also narrow close to $\eta _{} = 0.5$ as the range of possible $\eta _{}$ is restricted to the interval [0,0.5].

Predictions are improved by combining observations of the spatial statistics (Fig. 4b): the predicted values of $\rho$ are more accurate and the confidence intervals associated with the predictions are smaller and more consistent over a range of values of $\rho$. For example, while $\eta _{J}$ is the least accurate predictor of the single spatial statistics ($R^2 = 0.3033$), combining observations of $J_\text {min}$ and $g_\text {max}$ or $F_\text {max}$ causes the 95% confidence intervals associated with high values of $\eta _{}$ to become narrower. The most accurate classifier combining two statistics is $\eta _{g,F}$, implying that combining the highest performing spatial statistics may result in a better performing classifier. We note that $\eta _{F,J}$ performs worse than $\eta _{F}$ and that as such care must be taken when combining observations of spatial statistics to ensure that the resulting classifier is improved. Combining observations of all three statistics in $\eta _{g,F,J}$ yields the highest $R^2$ value ($R^2 = 0.8124$), and confidence intervals which are more consistent across the whole range of $\eta _{g,F,J}$ than for other $\eta _{}$ (mean widths of 95% CI: $\eta _{g} = 0.256, \eta _{F} =0.330, \eta _{J} =0.260, \eta _{g,F} =0.185, \eta _{g,J} =0.197, \eta _{F,J} = 0.268, \eta _{g,F,J} = 0.171$). Further, observations far from the line $\eta _{g,F,J} = \rho {}$ typically have wider confidence intervals than more accurately predicted values.

### Automated labelling of histological regions

**Figure 5:**
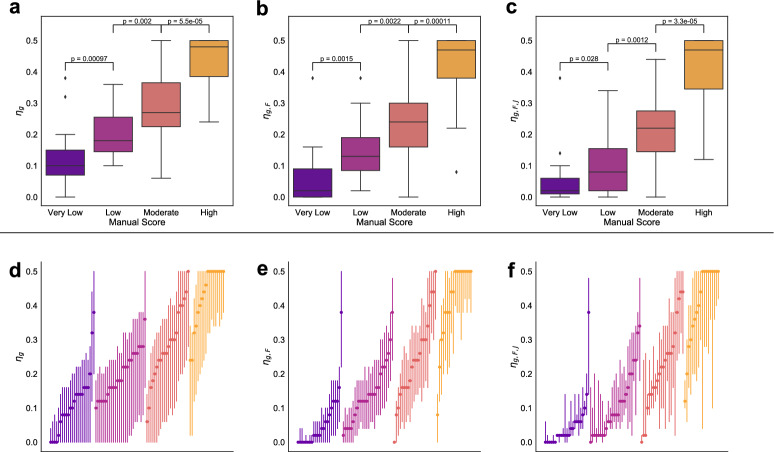
(**a**–**c**) Predicted values of $\eta _{g}$, $\eta _{g,F}$ and $\eta _{g,F,J}$ for the 89 manually scored head and neck ROIs. *p*-values determined using a two-sided *t* test and shown for adjacent boxplots only. (**d**–**f**): 95% confidence intervals (lines) for manually scored head and neck ROIs, for $\eta _{g}$, $\eta _{g,F}$ and $\eta _{g,F,J}$ (points). In each subplot, ROIs are ordered according to increasing $\eta _{}$, and coloured according to the manual score.

The seven metrics $\eta _{X}$ were applied to the 89 manually assessed head and neck ROIs. Figure 5a–c shows the resulting distributions of $\eta _{g}$, $\eta _{g,F}$ and $\eta _{g,F,J}$, partitioned by manual score of CD68+ macrophage infiltration. Each classifier identifies statistically significant differences between the manual scoring categories. We note that the pathologist assigns discrete scores to each image whereas $\eta _{}$ is a continuous quantity. Consequently patterns which lie at class boundaries may be difficult to score. This may explain some of the overlap in predictions of $\eta _{}$ between classes. Other extreme predictions are more clearly outliers; the ROIs corresponding to these images are examined in the Supplementary Information, Sect. J.

We note that the ability of $\eta _{g,F,J}$ to distinguish between manual scores is not significantly greater than the ability of $\eta _{g,F}$ or $\eta _{g}$. However, in line with Fig. 4, the confidence intervals associated with $\eta _{}$ become narrower as observations of additional statistics are combined.

Figure 5d–f shows 95% confidence intervals around $\eta _{}$ for the three classifiers, coloured according to their manual score and ordered in increasing value of $\eta _{}$. While Fig. 5a–c may suggest that $\eta _{g}$ or $\eta _{g,F}$ most successfully distinguish between different manual categorisations, Fig. 5d indicates that predictions made by $\eta _{g}$ tend to have wide confidence intervals. Combining statistics can reduce the width of the confidence intervals, as for $\eta _{g,F}$, but as shown in Fig. 4, may also increase their width. Incorporating observations of $J_\text {min}$ into $\eta _{g,F,J}$ eliminates low values of $\eta _{}$ from the confidence intervals, suggesting that there may be benefits from the inclusion of spatial statistics which do not appear promising in their own right.

A similar analysis for the locations of CD8+ cells within the head and neck cancer dataset can be found in the Supplementary Information, Sect. K.

## Discussion

Although pathologists’ training allows them to accurately assess immune cell distributions within tumours, it is infeasible manually to assess large numbers of images in a high throughput manner. Additionally, human assessment is qualitative or, at best, semi-quantitative in nature. Therefore, such a task requires the adoption of carefully calibrated digital image analysis and statistical approaches. Once accurate cell locations have been identified, application of spatial statistics can provide quantitative information about their spatial structure. Whilst individual spatial statistics may identify features such as clustering, it is less clear how these features relate to human descriptions of the complex patterns of immune cell distributions.

In this study, we investigated whether spatial statistics, individually or in combination, agreed with pathological assessment of macrophage distributions within images of human head and neck cancer slides. We identified three summary features ($g_\text {max}$, $F_\text {max}$ and $J_\text {min}$) which vary predictably with the distribution of macrophages. Although each metric correlates with increasing infiltration, there is substantial overlap in the ranges of these metrics and the semi-quantitative categories assigned by the pathologist. We conclude that while observation of a single metric is insufficient to discriminate between semi-quantitative categories assigned by a pathologist, it may be possible to discriminate between them by combining multiple statistics. In statistics and machine learning, including additional parameters in a model may cause overfitting, particularly when the same dataset is used for both testing and validation. While techniques exist to penalise models for overfitting based upon the number of parameters they consider^46,47^, in this manuscript the evaluation of the models is performed on an independent test set, which prevents selection bias and means that model complexity need not be explicitly accounted for when determining predictive accuracy. Introducing observations of additional statistics provides a more complete description of the point data, rather than causing overfitting due to increased model complexity.

Since manual assessment of histology images is time-consuming, it is difficult to leverage enough data to enable machine learning approaches. We therefore created synthetic datasets, designed to resemble those encountered in human samples but for which point dispersal is quantified by the parameter $\rho$. We used synthetic training data to determine the probability that a point pattern generated with a specific value of $\rho$ would give rise to particular values of $g_\text {max}$, $F_\text {max}$ and $J_\text {min}$. These probabilities allow estimation of $\rho$ from a given point pattern via MLE, based on observations of one or more of $g_\text {max}$, $F_\text {max}$ and $J_\text {min}$. In this approach, self-weighting ensures that a given metric contributes more to the likelihood function in images where its probability distribution is more informative. Predictions based on individual spatial statistics were not strongly predictive, but were improved when two spatial statistics were used, and were best when observations of all three statistics were combined. Using multiple statistics also reduced the width of the associated confidence intervals. The ability of MLE to provide an estimate of error for each prediction could also be used to flag images with wide confidence intervals for pathologist review. Furthermore, these descriptors can also flag for human review images with low numbers of cells and/or large regions devoid of immune cells, a common finding in images affected by missing tissue or other histological artefacts (see Supplementary Information, Sect. I). Finally, when applied to the original histology images, several MLE predictions, based on different combinations of the three spatial statistics, could distinguish between pathologist assigned categories and the associated confidence intervals became narrower when the statistics were combined. Although the ability of $\eta _{g,F,J}$ to distinguish between manual scores is not significantly increased compared to $\eta _{g}$ and $\eta _{g,F}$, there is less uncertainty in evaluating $\eta _{g,F,J}$ as estimations are more precise and have narrower confidence intervals. Importantly, we do not claim that $g_\text {max}$, $F_\text {max}$ and $J_\text {min}$ are the optimal statistics to observe in order to best predict pathologist scores. Instead, the value of this work is as a proof-of-concept approach in which multiple statistical descriptions of a point pattern are combined to produce a single numerical description which coincides with qualitative evaluation of the point pattern.

Our training data can be expressed as a high-dimensional list of features, and therefore used to train classifiers using machine learning techniques. An advantage of using MLE over these techniques is that it is straightforward to estimate confidence intervals around $\eta _{}$. Further, as spatial statistics describe features such as clustering or dispersion, metrics derived from their combinations can be understood in terms of the point cloud structure. Interpretation of decisions made by our approach is therefore simple compared with black-box algorithms such as neural networks where key features are often poorly understood.

The method introduced in this paper is generic in the sense that it is not tied to the particular spatial statistics employed; it can easily be extended to incorporate different spatial statistics. Possible alternatives, which have been applied to digital pathology data, include the Morisita-Horn index of colocalisation^23,24,48^, Getis-Ord hotspot analysis^26,49^ and combinations of morphological characteristics^22^. Other statistics designed to characterise immune infiltration in histological data include the intratumour lymphocyte ratio^27^, which measures the ratio of the number of intratumour lymphocytes and the number of tumour cells, and the Immunoscore^28–30^. Including observations of these statistics may improve predictions of pathologists’ categories, and could be tested by generating synthetic validation data following our method. Importantly, adding additional statistics does not always lead to a more accurate prediction. Care must therefore be taken when selecting spatial statistical descriptors to ensure improvement. This is most likely to be obtained by using a range of statistics which identify different features of point clouds, so choosing statistics based on their function rather than making arbitrary decisions is likely to yield the best descriptors. Future work using our method will identify an optimised set of statistics which better describe immune infiltration.

Our method is not reliant on the process used to generate synthetic data; alternative processes which generate point clouds resembling immune cell distributions could be used. This flexibility means that our approach for combining spatial statistical observations could be applied more widely, given an appropriate method for generating synthetic training data. Examples of histological investigations where this may be useful include quantifying colocalisation of different immune cell subtypes, or describing relationships between immune cells and the tumour vasculature.

## Supplementary information

Supplementary Information.

## Data Availability

Code providing a working example of our image analysis scripts can be found at https://github.com/JABull1066/ImageAnalysisScripts. Code and data relating to combining spatial statistics and reproducing results in this manuscript can be found at https://github.com/JABull1066/CombiningSpatialStatistics.
