# Supplementary material for: Combining multiple spatial statistics enhances the description of immune cell localisation within tumours
Source: Sci Rep. 2020 Oct 29;10:18624. doi: 10.1038/s41598-020-75180-9 (PMC7596100; doi:10.1038/s41598-020-75180-9)
Supplement: Supplementary file 1 — Supplementary Information. [file 41598_2020_75180_MOESM1_ESM.pdf]

Supplementary Information - Combining multiple spatial statistics enhances the description of immune cell localisation within tumours

Joshua A. Bull[1,*], Philip S. Macklin[2], Tom Quaiser[3], Franziska Braun[3], Sarah L. Waters[4], Chris W. Pugh[2], and Helen M. Byrne[1]

[1]Wolfson Centre for Mathematical Biology, Mathematical Institute, University of Oxford, Oxford, OX2 6GG, UK

[2]Nuffield Department of Medicine, NDM Research Building, University of Oxford, Oxford, OX3 7FZ, UK

[3]Roche Pharma Research and Early Development, Roche Innovation Center Munich, pRED Informatics, Nonnenwald 2, 82377 Penzberg, Germany

[4]Oxford Centre for Industrial and Applied Mathematics, Mathematical Institute, University of Oxford, Oxford, OX2 6GG, UK

[*]joshua.bull@maths.ox.ac.uk

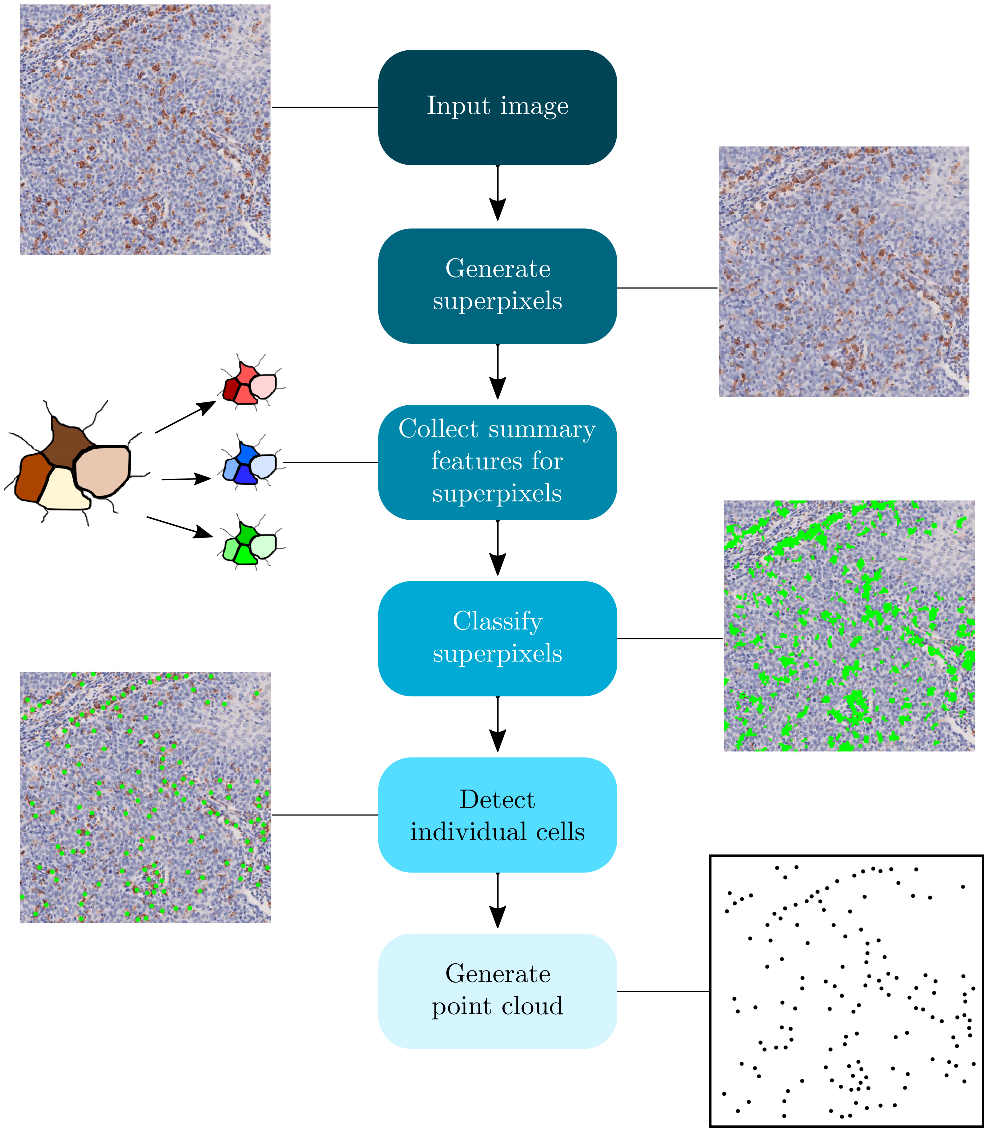

Figure A.1. Structure of our image analysis pipeline. Stages are separated to maximise the flexibility of the pipeline: alternative methods for superpixellation, classification or cell detection can be easily substituted if desired.

A Image analysis pipeline

In this Section, we describe the image analysis pipeline that we use to identify individual immune cells within regions of interest (ROIs) on IHC images and to represent them as point clouds. A schematic of the workflow is presented in Figure A.1. We note that many alternative approaches may be used to extract such point clouds and that the modular nature of our workflow renders their substitution into the pipeline straightforward. Further, the methods used in the image analysis pipeline do not affect the spatial statistical analysis used to characterise the immune cell point clouds.

A.1 Input image

The resolution of the histological slide scans is typically 0.2205 microns per pixel (40x effective magnification). Consequently, a tumour section of 3.5 cm $\times$ 2 cm may generate an image containing approximately 1.4×10^{10} pixels. This presents a challenge for image analysis algorithms, which must classify each pixel as positively or negatively stained. We conduct image analysis on lower resolution images of approximately 0.882 microns per pixel (10x effective magnification). The images we analyse are ROIs cropped from whole slide images. To further increase analysis speed, whole slide images can be decomposed into "tiles" of approximately 1000×1000 pixels which can be analysed in parallel using multiple computational cores.

Our pipeline permits user annotations of images. These can be imported from other software as an XML file, or applied using MATLAB's inbuilt annotation tools. Annotations can be used to exclude areas from the classification (e.g. surgical ink which could be confused with IHC staining, or areas of damaged tissue or necrosis). Annotations are used to guide ROI selection; the tumour boundary for each case was annotated by a pathologist, and square ROIs selected randomly, ensuring that ROIs do not overlap and/or cross annotated tumour boundaries. For each tumour, ROIs were continually sampled until the annotated region was saturated.

A.2 Superpixel generation

Our pipeline uses superpixels to reduce image complexity while retaining object boundaries[1]. Superpixels are clusters of adjacent pixels with similar colour intensities. They have been used to identify morphological features of breast cancer in histological images[2,3] and to preprocess histological slides for further analysis[4]. A wide range of superpixellation algorithms exist, providing varying degrees of control over properties such as their size and shape (for a comprehensive review, see Stutz et. al[5].) We use the SLIC (Simple Linear Iterative Clustering) algorithm[6], a fast algorithm which provides control over the size of superpixels and generates superpixels which adhere well to the boundaries of objects in images[5]. We generate an oversegmented image using superpixels with an area of approximately $20\mu m^2$.

A.3 Collect summary features for superpixels

We collect a range of summary features which describe the colour and shape of each superpixel and its neighbouring superpixels (those which share an edge). These features are used to train feature-based classifiers. The SLIC algorithm operates in the CIELAB colour space, where the colour of each superpixel is described by the channels L, a and b. We use the mean and variance of each channel, and those of the neighbouring superpixels, to define summary features for each superpixel. The features collected for each superpixel are shown in Table 1.

A.4 Classify superpixels

A binary support vector machine (SVM) is applied to labelled training data to locate a hyperplane in the high dimensional feature space which optimally separates positively and negatively stained superpixels[7]. Superpixels are then assigned to a class based on their location in feature space relative to the hyperplane. SVMs have previously been used to segment a range of biomedical images including electron microscope images[8,9], mammogram images[10] and magnetic resonance (MR) images[11].

Number	Feature
1-3	Mean of L, a, b channels for superpixel
4-6	Variance of L, a, b channels for superpixel
7-9	Mean of L, a, b channels for neighbouring superpixels
10-12	Variance of of L, a, b channels for neighbouring superpixels
13-15	Mean of L, a, b channels for neighbours of neighbours
16-18	Variance of L, a, b channels for neighbours of neighbours
19-21	Superpixel width, height and aspect ratio
22-24	Area, perimeter, and perimeter/area ratio of superpixel
25-26	Number of neighbours / neighbours of neighbours

Table 1. Features used for SVM classification of superpixels.

They have also been used for cell detection in histology images[12,13]. By combining binary SVMs, our pipeline can be extended to identify multiple stains on an image.

A.5 Detect individual cells

We use a watershedding algorithm to identify distinct objects in positively classified regions of pixels, although alternative algorithms can also be used if desired. Alternative cell segmentation algorithms that could be used in place of watershedding include those based on morphological operations such as opening or closing, Laplacian of Gaussian filtering, level sets, or convolutional neural networks (for a comprehensive review, see Xing *et al*[14]). We use watershedding here because it has been shown to perform well at cell counting tasks, and is both fast and simple[13,15,16].

Cell detection proceeds as follows (see also Figures A.2(b-e)). First, noise is removed from the classification mask by removing all connected components with an area less than $25\,\mu m^2$. A distance transform is then applied to the mask before watershedding is used to separate the mask into discrete connected components. Connected components smaller than $56.25\,\mu m^2$ are removed, and any components larger than $1,225\,\mu m^2$ are divided in two (these areas are equivalent to squares with edge lengths $7.5\,\mu m$ and $35\,\mu m$ respectively). This process is repeated until all connected components are within the specified size range. The predicted cell centroids are returned as the centroids of the connected components. The thresholds described in this section were determined by a pathologist as best identifying CD68+ cells in our images; different thresholds can be used to identify other cell types.

Watershedding produces a labelled segmentation of individual cells. As in Figure A.2, cell centres are then determined by placing a point at the minimum of each watershed basin. The resulting point cloud lists the (x, y)-coordinates of each cell centre.

A.6 Fast generation of training data for new classifiers

To classify cells in an image, an appropriately trained classifier must be specified. The modular nature of our pipeline (Figure A.1) enables alternative classifiers to be easily used. A graphical user interface (GUI) enables new classifiers to be trained from representative images (see Figure A.2). This interface allows fast selection of positively stained pixels, which are then used to train and optimise the classifier. Training images are presented in both high resolution and as superpixellated

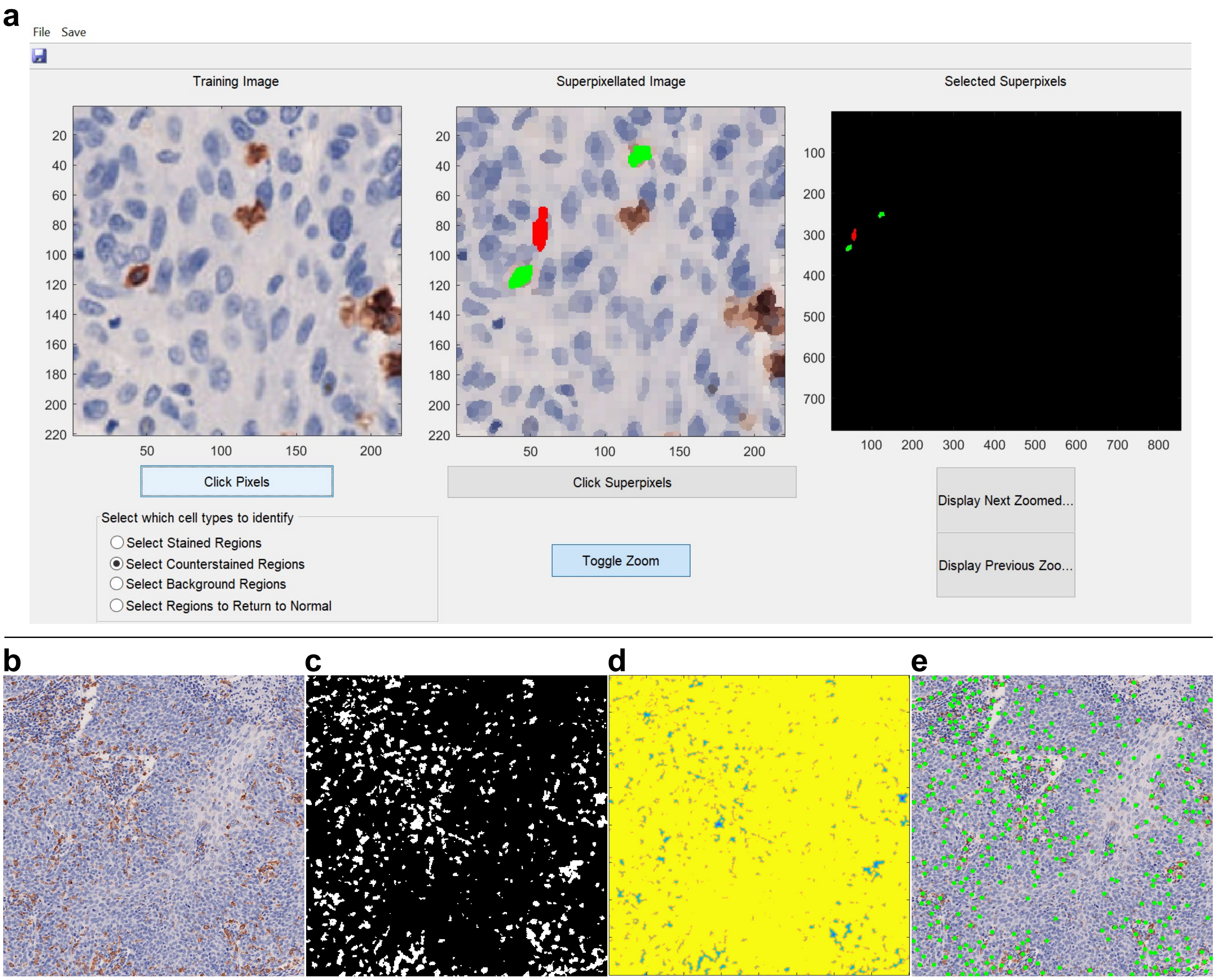

Figure A.2. (a) Example of using our pipeline's graphical user interface (GUI) for fast training of new classifiers from representative image sections. Users can assign superpixels to different classes by selecting relevant pixels from the high-resolution image. The classifiers shown in this paper were trained on between 4 and 12 randomly selected snapshots from different histology slides to account for variability in staining. (b) Section of initial image. CD8+ cells are shown in brown; (c) CD8+ staining identified by a classifier; (d) Distance transform for watershedding-based segmentation of CD8+ cells; (e) Predicted CD8+ cell centres (minima of watershedding catchment basins), shown as green dots.

Classifier Name (SVM)	PM	SJ	AY	Combination
CD8 training data size (superpixels)	7951	2074	1580	11605
CD68 training data size (superpixels)	8694	1877	787	11358
CD8 number of support vectors	327	219	221	602
CD68 number of support vectors	303	221	213	607

Table 2. Number of superpixels labelled as training data and number of support vectors for each classifier.

versions, and users can click on positively stained cells using either high resolution or superpixellated images.

Our pipeline and user interface are currently implemented within MATLAB, although a standalone implementation is planned. Many of the algorithms described above are implemented in C via MEX files (e.g. for superpixellation and feature collection).

B Performance and validation of image analysis pipeline

In Figure B.1(a) we show receiver operating characteristics (ROC) for four SVM classifiers based on training data obtained by three people with varying degrees of experience in pathology (PM, SJ, AY) who were first time users of the software. We demonstrate classifiers for identifying two different cell types: CD68+ macrophages, and CD8+ T cells. The performance of each classifier was compared against a testing dataset of labelled images generated by a pathologist (PM). Each classifier performs well, with an area under the curve (AUC) of greater than 0.96. Table 2 details the number of superpixels labelled for training data for each classifier, together with the number of these superpixels used by the resulting classifier as support vectors. The "combination" classifier was trained using all of the training data provided by the three volunteers. These results show that an accurate classifier can be trained by users who lack experience of the software.

Figures B.1(b-e) show further validation of the "combination" classifier in a real world cell counting task. Forty randomly selected 1.5 mm × 1.5 mm regions were chosen from CD8+ and CD68+ head and neck cancer IHC slides and the number of positively stained cells in each region was counted by three human volunteers, with one volunteer counting the cells in the images on two occasions separated by several months. Figure B.1(b) shows the intra-observer variation in the reported cell counts. The greatest variation is within regions of CD68+ images regions that contain many cells. Figure B.1(c) shows that the counts made by our pipeline (SIGHT) are consistent with the human counts. Figure B.1(d) shows examples of the regions used in the cell counting task, and indicates the mean time taken for cell counting in each image. We note that human counts are less reproducible and slower than automated cell counts. Figure B.1(e) shows the mean and range of the human counts for each region (log plots) compared with three different automated systems. Our pipeline (SIGHT) most accurately reproduces the human counts. Classifiers for ImageJ[17, 18] and Visiopharm (`https://www.visiopharm.com/`) were trained by local experts in the software. While each algorithm produces the same trend, cell counts made by our algorithm fall within the range of counts made by humans most frequently.

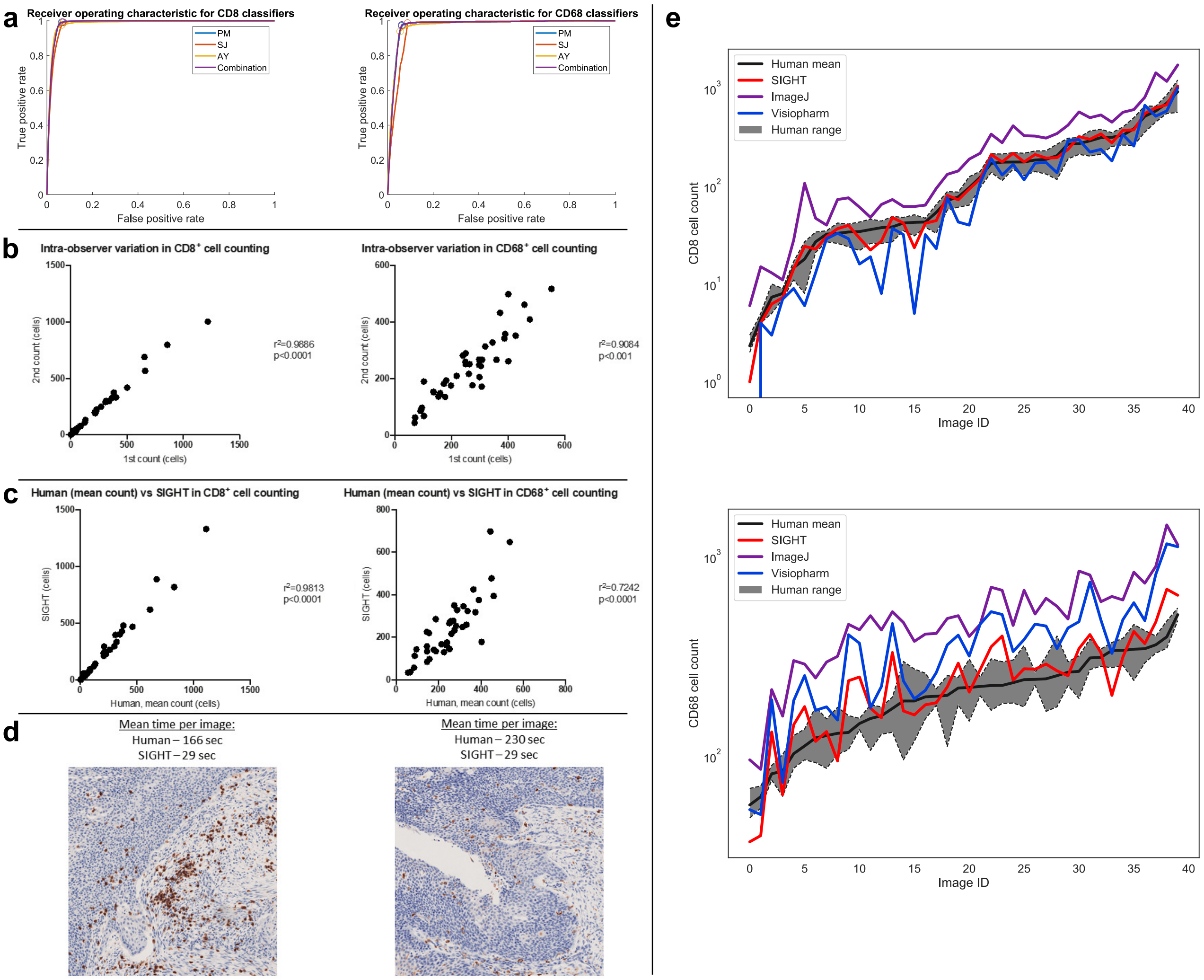

Figure B.1. (a) Receiver operating characteristics for SVM classifiers for detection of CD8+ and CD68+ cells trained by three different people (PM, SJ and AY) using our framework, and for an SVM classifier combining the training data from each of these classifiers. AUC: CD8 - PM=0.984, SJ=0.980, AY=0.982, Combined=0.984. CD68 - PM=0.973, SJ=0.961, AY=0.970, Combined=0.972. Optimum thresholds used for classification are marked with a circle. (b) 3 human volunteers counted the number of CD8+ and CD68+ cells in 40 randomly selected 1.5mm x 1.5mm regions chosen from head and neck tumours, with one volunteer assessing the same images on two separate occasions. The intra-observer variation in the number of cells reported for each image is shown for both CD8+ cells and CD68+ cells. Observers were reasonably consistent in the number of reported cells, with more variation for CD68+ cells and for images with more cells. (c) Comparison between cell counts for the human volunteers (mean) and our imaging pipeline (SIGHT) for both CD8+ and CD68+ cells. (d) Example images used in the cell counting tasks for CD8+ (left) and CD68+ (right). Human cell counting takes much longer than machine counting. (e) Number of CD8+ and CD68+ cells counted in each region by humans (mean: black line, range: grey area) and by three different automated cell counting protocols (log plot). Image IDs are ordered according to mean human cell count. Our pipeline (SIGHT) is closest to the human counts. Classifiers trained by local experts in ImageJ[17,18] and Visiopharm (https://www.visiopharm.com/) report counts which are generally either over- or under-counting. For all classifiers, any error from the human counts appears to grow exponentially.

C Calculation of the PCF

In this Section, we provide additional description on how boundary conditions are implemented for the PCF. In calculating the SCD and J-function there is no need to compensate for boundary effects, but there is often overlap between the edge of the domain and the annuli used in calculating the PCF. Such effects must be taken into account when calculating the PCF[19].

When an annulus intersects the boundary of a region of interest, its contribution to the PCF must be appropriately scaled according to its area. Consider the annulus of radius r and width dr around a point i. We calculate the area of the annulus, $A_{i,r}$, which is contained inside the domain. If point i is further than $r+dr$ from the domain boundary, then this annulus will be entirely contained within the domain and hence $A_{i,r} = \pi\left((r+dr)^2 - r^2\right)$. For points which are closer to the edge of the domain than this distance, the annulus will intersect with the domain boundary and will therefore have a smaller area, which must be calculated separately for each annulus.

If the area of the domain is A and the point pattern contains n points, then the average density of points is $d = \frac{n}{A}$. The expected number of points in an annulus under complete spatial randomness (CSR) is therefore $n_{\text{CSR}} = d \times A_{i,r}$. If the annulus of radius r around point i contains n_{i} points, then we calculate $g_i(r) = n_{\text{i}}/n_{\text{CSR}}$. The PCF, $g(r)$, is then calculated by averaging this over every cell i, $g(r) = \sum_{i=1}^{n} g_i(r)/n$.

D Generation of simulated immune cell distributions

In addition to the point clouds extracted from IHC images, the second source of point patterns we consider is synthetically generated immune cell distributions. These point patterns are used to generate a training dataset, with which we evaluate how spatial statistics vary along with the parameter ρ, and a testing dataset, which we use to evaluate how well MLE can predict the value of ρ used to generate a point cloud. In this Section, we describe the process which we use to generate these point patterns. The key biological phenomenon which we wish to simulate is the infiltration of one type of immune cell into clusters of tumour cells embedded within stromal regions, so the synthetic data should emulate this. Simulated immune cell distributions are therefore generated under the following assumptions:

- Immune cell centroids are separated by a minimum distance, which represents the approximate diameter of an immune cell. We fix this distance to be $20\,\mu m$.
- The difference between an immune-rich and an immune-poor region (saturated or unsaturated tumour cell nests) is determined by the relative density of immune cells in each region. Thus an image contains an immune desert if the immune cell density in one part of the image is very low relative to the density in another part.

We generate synthetic point clouds by first generating synthetic tissues that comprise two distinct regions which we term “stroma” and “tumour.” These regions are obtained by using Gaussian random fields (GRF)[20] to create a binary mask with a dominant length scale, l (Figure D.1). The GRF is created in the Fourier domain by specifying the size of the desired region (we use a 1.5 mm $\times$ 1.5 mm square for all simulated point patterns) and placing a peak at the frequency corresponding to the

desired length scale l. Random noise is added in Fourier space. The spectrum is then inverted and the imaginary part discarded. This process reproducibly produces random fields with different characteristic length scales, that mimic a range of tumour geometries. The mask is binarised by thresholding the random field. We reject binary masks in which either of the regions, tumour or stroma, occupies less than 25% of the ROI.

Once the binary mask has been generated, point clouds are generated by first calculating the total areas of the stromal and tumour regions, A_s and A_t and prescribing two parameters: d, the target density of points in the domain, and ρ, the relative density of points in the tumour and stroma regions. Given A_s, A_t, d and ρ , we determine the target number of points for the stromal and tumour regions by noting the following identities:

$$d = \frac{n_s + n_t}{A_s + A_t} = \frac{n_{TOT}}{A_s + A_t}, \tag{1}$$

and

$$\rho = \left(\frac{n_t}{A_t}\right) / \left(\frac{n_s}{A_s}\right), \tag{2}$$

where $n_{TOT} = d\,(A_s + A_t)$ is the total number of points in the domain. These equations can be rearranged to define n_s and n_t in terms of A_s, A_t, d and ρ:

$$n_t = \frac{n\rho \frac{A_t}{A_s}}{1 + \rho \frac{A_t}{A_s}} \quad \text{and} \quad n_s = n_{TOT} - n_t \tag{3}$$

If new points cannot be placed in one region and the target density has not been reached, cells are removed from the other region and the process repeated until the target number of cells have been distributed across the domain. The final values of ρ and d are then recorded to ensure that the simulated point pattern has the desired properties. Figure D.2 shows how, for a given tumour geometry, varying ρ and d affects the point clouds that are generated. We note that when $\rho = 0$ then the tumour regions are immune deserts, devoid of points, and that when $\rho = 1$ there is no immune cell exclusion (points are uniformly distributed across the domain).

There are many other ways to generate synthetic point clouds, including cluster processes. For example, Cox point processes use ‘parent’ points to seed secondary clusters, with differences in the distributions of parent and daughter points giving rise to Poisson, Matérn, Neyman-Scott, and Modified Thomas cluster processes[21]. While such processes could be used to generate point clouds representing immune cell infiltration, since we aim to characterise the distribution of immune cells within tumour and stroma regions, we use the process outlined for point placement as it distinguishes between the two regions.

Figure D.3 shows the effect of varying the length scale parameter l on the structure of point patterns. It shows three synthetic geometries, generated with length scales (a) $l = 0.5$ mm, (b) $l = 0.25$ mm and (c) $l = 0.1$ mm in a domain of side length 1.5 mm. Geometries with large l (e.g., Figure D.3(a)) create broad areas of tumour and stroma with relatively smooth edges. As the

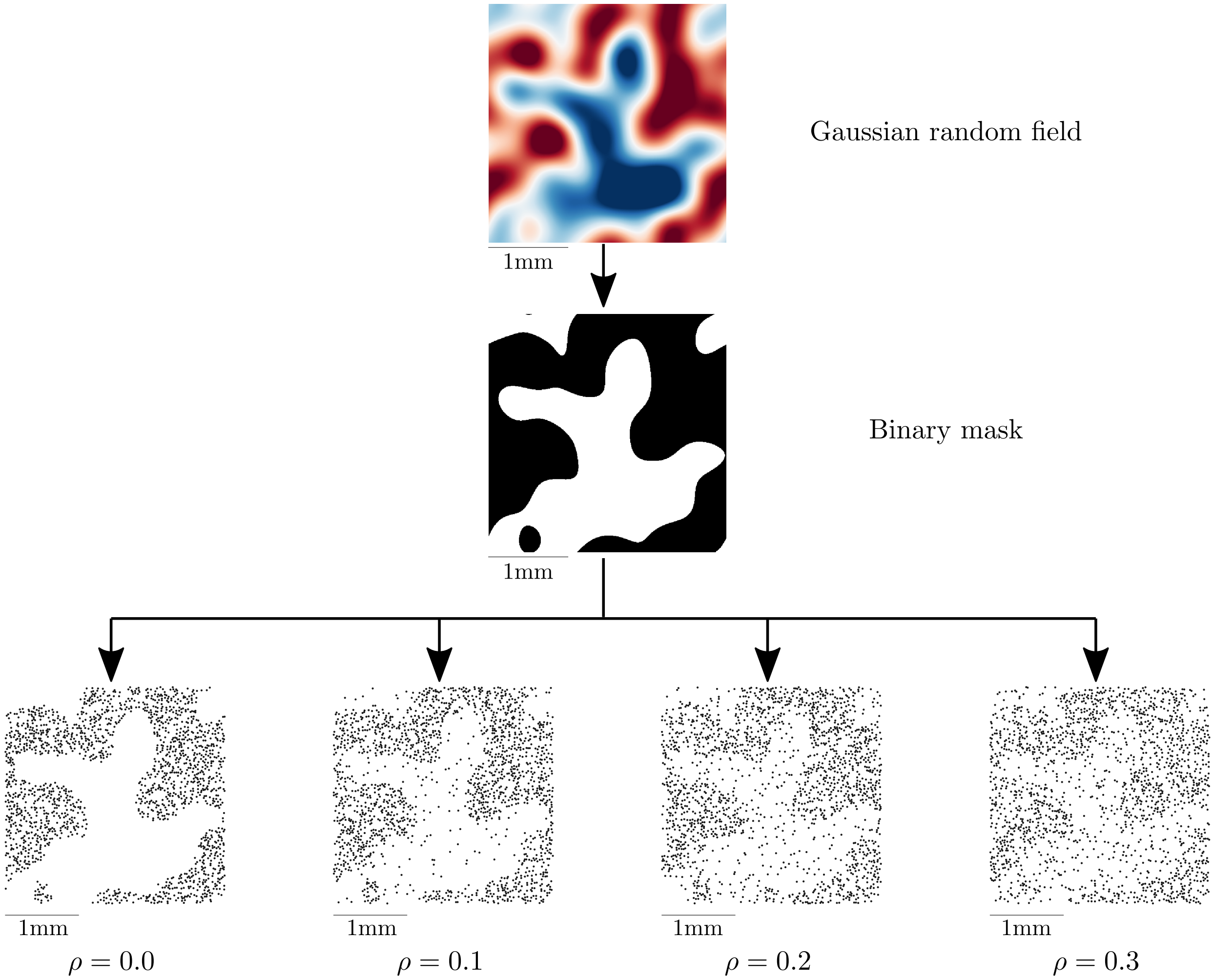

Figure D.1. Stages in generating an artificial immune cell distribution within segregated tumour and stroma regions using Gaussian random fields. A Gaussian random field is generated with a fixed characteristic length scale ($l = 0.5$ mm). Tumour (white) and stroma (black) regions are defined by thresholding the random field to produce a binary mask. Regions where the field is positive are classed as stroma, while those where the field is negative are classed as tumour. Cells are then placed with a specified density (here 200 cells per mm^2). The areas of the tumour and stroma are used to identify how many points are required in each in order to maintain the desired ratio of cells ρ. Points are then randomly selected from across the image, and are added to the point pattern if the number of cells in its region (tumour/stroma) has not yet been reached and they do not fall within the exclusion radius of another point.

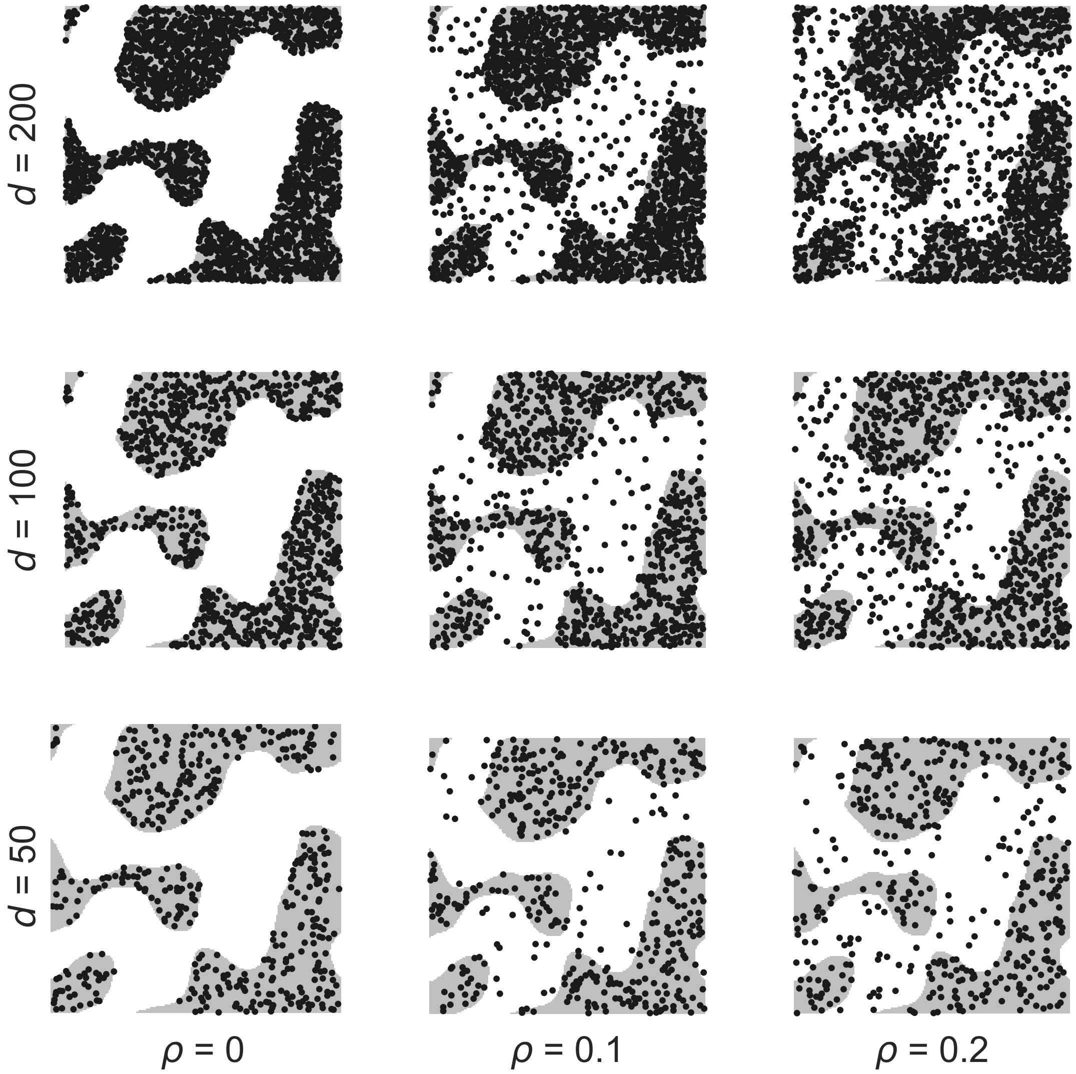

Figure D.2. Series of images showing how typical simulated immune cell infiltration patterns into the same virtual tumour cell nest change as the immune cell density d (cells per mm^2) and the ratio of cells in the tumour cell nest to the stroma, ρ, vary. The white region corresponds to the tumour cell nest and the grey region to the stroma. These infiltration patterns illustrate that cell density alone does not capture immune cell localisation in tumour cell nests and/or stroma, information which can be simulated by the parameter ρ.

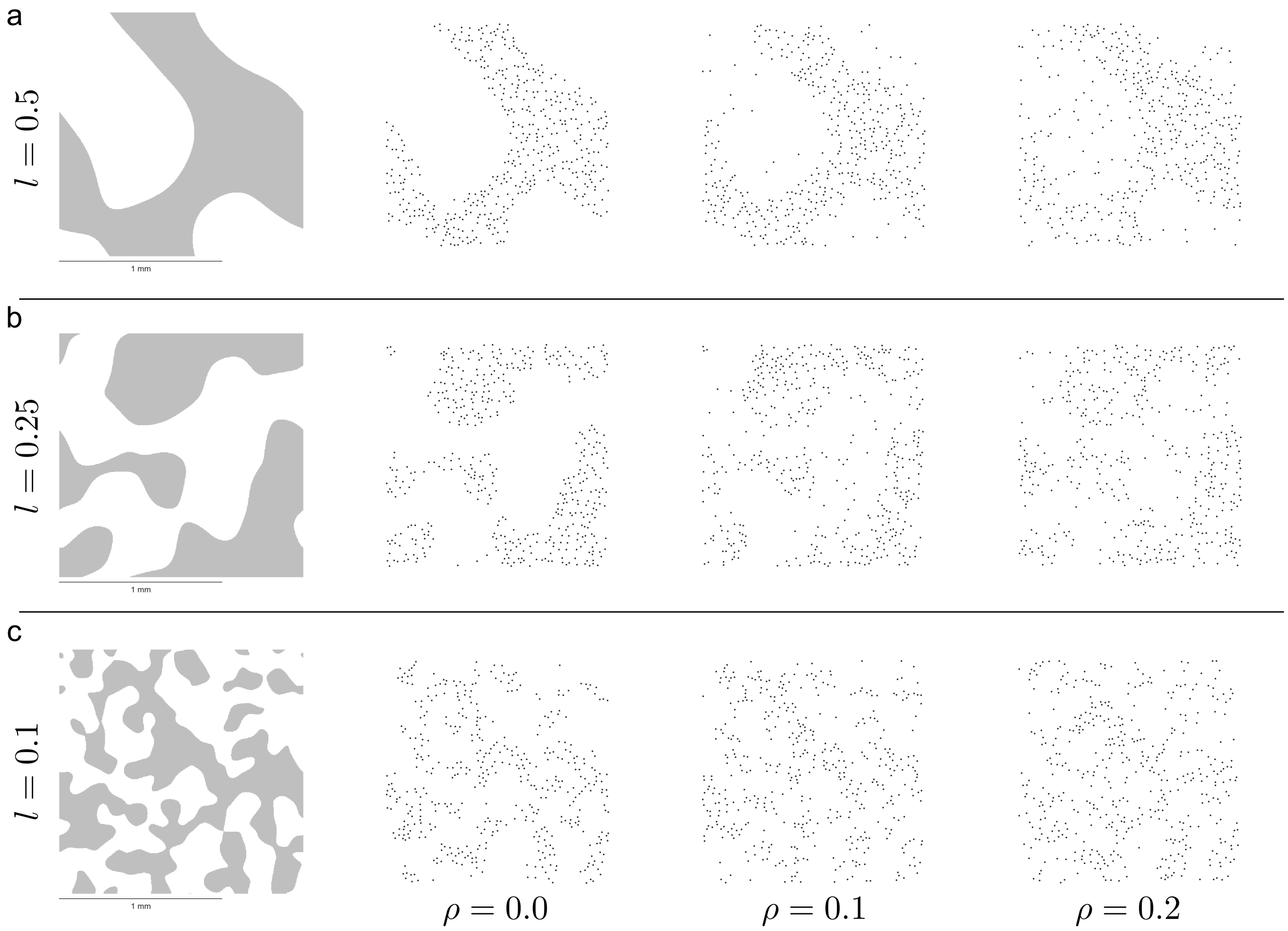

Figure D.3. Series of images showing how varying the length scale l alters the geometry of the simulated stroma and tumour cell nest. The white region corresponds to the tumour cell nest and the grey region to the stroma. Variations in the length scale must be considered when defining infiltration, as it is difficult to distinguish by eye between a heavily infiltrated tumour nest with a large length scale (e.g., $l = 0.5, \rho = 0.2$) and a tumour cell with low infiltration but a convoluted structure (e.g., $l = 0.1, \rho = 0$).

length scale decreases, the edges of the pattern become less smooth and there are no longer wide regions of either tumour or stroma. This models variation within tumour nests, which can be convoluted or compact.

E Synthetic data with different parameter ranges

We impose restrictions on parameters used to generate synthetic point patterns. In particular, we consider only point patterns in which $\rho \in [0, 0.5]$ and $d \geq 150$ cells per mm^2. Figures E.1 and E.2 reproduce the results of Figure 4 when both training and testing datasets contain point patterns with $\rho \in [0, 1]$ and d is unrestricted.

ρ describes the relative density of points between tumour cell nests and stroma. One strength of our method is that it is based only on the (x, y)-coordinates of point patterns, and therefore does not require identification of the tumour/stroma boundary to be applied to IHC data. As a proxy for this, we assume that macrophages are relatively more dense in the stroma

and that higher local cell density can therefore be used to approximate the tumour/stroma boundary. Under this assumption, the maximum possible range of ρ is [0,1], where $\rho = 1$ implies that macrophages are equally represented in both regions. However, as ρ increases it becomes difficult for human assessment to distinguish between point patterns - all synthetic patterns appear to contain highly infiltrated tumour nests. As patterns with $\rho > 0.5$ appear homogeneous, we exclude this data to ensure that the testing and training datasets remain perceptually balanced between patterns with low and with high infiltration.

Maintaining a difference in local point density also motivates our restriction of $d \geq 150$ cells per mm^2. We use the local cell density to distinguish between stroma and tumour. However, when d is low the local density in both tumour and stroma will be low. For sufficiently low overall d, this makes it impossible to distinguish between tumour cell nests and stroma based only on the point pattern. The statistics we consider in this report identify local clustering and spaces between cells, which are present in regions with large immune deserts, but also in those with overall low cell density. This is because for low d point patterns can be constructed with high ρ but which are unstructured, and whose spatial statistics are consequently similar to those of denser point patterns with low ρ. Figure E.2 shows that point patterns with low cell densities are typically predicted to have been generated with low ρ. We therefore limit the possible range of d used here, and note that point patterns with low cell density cannot be reliably classified using the three statistics considered here.

F Distribution of spatial statistics within Roche Immunohistochemical Tissue Atlas (RITA) data

F.1 Staining protocol for immunohistochemistry

We consider IHC slides from the Roche Immunohistochemical Tissue Atlas (RITA) dataset[22]. 4 μm sections were cut from formalin-fixed paraffin embedded tissue blocks of human breast, colorectal and prostate cancer (60 cases of each cancer type). These sections underwent manual immunohistochemistry staining. Briefly, sections were deparaffinized, underwent epitope retrieval (by heating for 10 minutes at 97-100 °C in a pressure cooker with pH 9.0 antigen retrieval solution [Thermo Fisher Scientific]) and endogenous peroxidase activity was blocked with 3% hydrogen peroxide (5 minutes; Merck, catalogue no. 107210). Next, sequential blocking of non-specific background staining (10 minutes; Agilent, catalogue no. X0909) and endogenous biotin (as per manufacturer's instructions; Agilent, catalogue no. X0590) was performed. Subsequently, sections were incubated overnight with the primary antibody in antibody diluent (Agilent, catalogue no. S2022). Next, sections were sequentially incubated with a biotinylated anti-mouse IgG secondary antibody (30 minutes) and then avidin-biotin complex (30 minutes; both reagents included within the VECTASTAIN Elite ABC-HRP Kit, Vector Laboratories, catalogue no. PK-6102). Finally, DAB chromogen was applied for 10 minutes (Agilent, catalogue no. K3468) and slides were counterstained with haematoxylin. The following primary antibody was used during staining: CSF-1R (mouse monoclonal, clone 29), Roche Diagnostics, Germany (in-house generated), 0.9 μg/mL concentration. The positive control sample comprised a section of CSF-1R transfected xenograft tumour tissue. Slides were scanned using an Aperio ScanScope slide scanner, and regions of interest (ROIs) were selected at 10x effective magnification. ROIs were obtained by saturating each tissue sample with

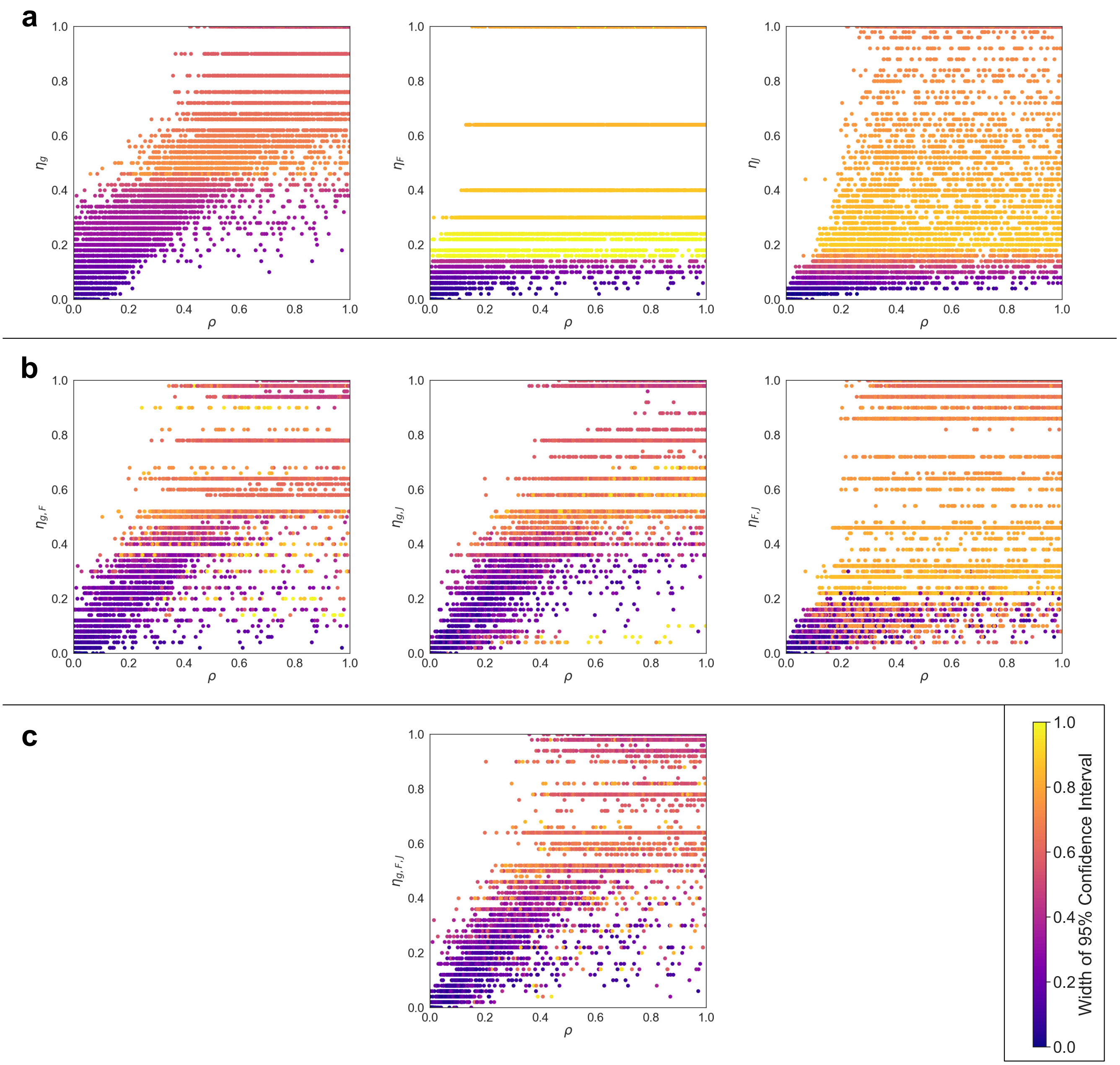

Figure E.1. Reproduction of Figure 4, with $\rho \in [0, 1]$ and without censoring of ROIs for which $d < 150$. R^2 values: (a) 0.709, 0.230, 0.366, (b) 0.681, 0.740, 0.382, (c) 0.710.

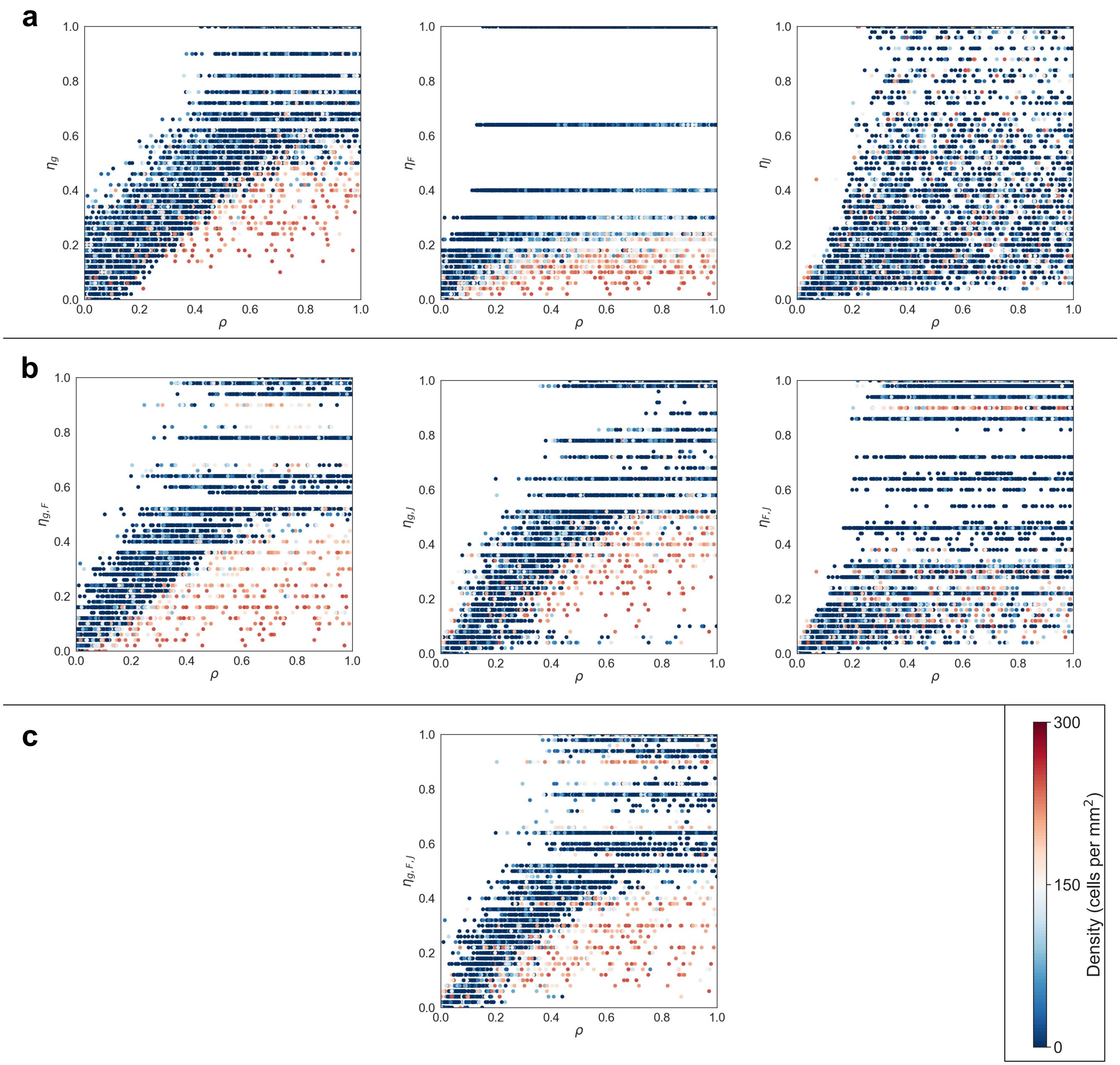

Figure E.2. Copy of Figure E.1 with ROIs coloured according to CD68+ macrophage density. Point patterns with $d < 150$ are shown in red, and those with $d > 150$ are shown in blue. Point patterns with low density are typically predicted to have low η.

non-overlapping squares, resulting in: 1,378 0.75 mm × 0.75 mm breast cancer ROIs, 429 3 mm × 3 mm colorectal cancer ROIs, and 1,208 1.5 mm × 1.5 mm prostate cancer ROIs.

F.2 Comparison of spatial statistics across different cancer types

The method developed in this paper can be applied to point patterns extracted from IHC samples arising from different tumour types. In this Section we calculate the spatial statistics ($g_{\max}$, $F_{\max}$ and $J_{\min}$) for 3,564 samples across four tumour types, comparing the head and neck ROIs described in the main paper with samples from the RITA dataset (549 head and neck, 1.5 mm × 1.5 mm; 1,378 breast, 0.75 mm × 0.75 mm; 429 colorectal, 3 mm × 3 mm; 1,208 prostate, 1.5 mm × 1.5 mm). The distributions and pairwise observations (correlations) of the macrophage density, $g_{\max}$, $F_{\max}$ and $J_{\min}$ are presented in Figure F.1.

The distributions of macrophage densities are comparable between the different types of cancer, although the head and neck ROIs tend to have lower macrophage density while the breast cancer ROIs exhibit a broader range of densities. The distributions of $g_{\max}$, $F_{\max}$ and $J_{\min}$ are similar for each cancer type, although there are subtle differences in the location of the peaks and the number of ROIs in the tails. While these differences between cancer types may be due to differences in macrophage distributions, they may be due to differences in the protocols used for data collection. For example, variations in the sizes of ROIs between samples may explain why the corresponding macrophage densities have a wider range for breast cancer samples and a narrower range for colorectal cancer samples. Furthermore, while the head and neck slides are stained for the macrophage marker CD68, the other cancer types are stained for the macrophage marker CSF-1R. Since the statistical distributions differ between cancer types, we suggest that in future work new synthetic data are generated to more closely resemble the distribution of spatial statistics observed in the particular cancer type being analysed.

Although the observations of $g_{\max}$, $F_{\max}$ and $J_{\min}$ shown here could be used to calculate η for the RITA data ROIs, there is no corresponding evaluation of macrophage infiltration available. Since there is no benchmark against which to compare the performance of η we omit this analysis.

G Effect of varying the length scale of the tumour cell nest and the immune cell density on spatial statistics

The synthetic data used for training and validation has randomly sampled length scale l and cell density d. l is used in point pattern generation when creating the binary mask distinguishing stroma and tumour cell nests. In this section, we show how varying l and d affects $g_{\max}$, $F_{\max}$ and $J_{\min}$ in synthetic data. Figure G.1 shows parameter sweeps in which l or d are held constant while ρ varies. In Figure G.1(a), we vary ρ and l while holding d constant at $d = 400$ cells per mm^2. This shows that varying l does not have a strong impact on $g_{\max}$ or $J_{\min}$, although there is increased variance in the range of $g_{\max}$ when ρ is small (e.g., $\rho < 0.2$). The main effect of varying l is in $F_{\max}$ when ρ is small. $F_{\max}$ measures the size of the largest void in the point pattern. When few points are placed in the tumour cell nest region (for ρ close to 0), the size of the largest void is determined by the width of the tumour cell nest region, which by definition is determined by l. In Figure G.1(b) we vary ρ and d while holding constant $l = 0.7$ mm. The most prominent difference is again in $F_{\max}$, where increasing d results in lower

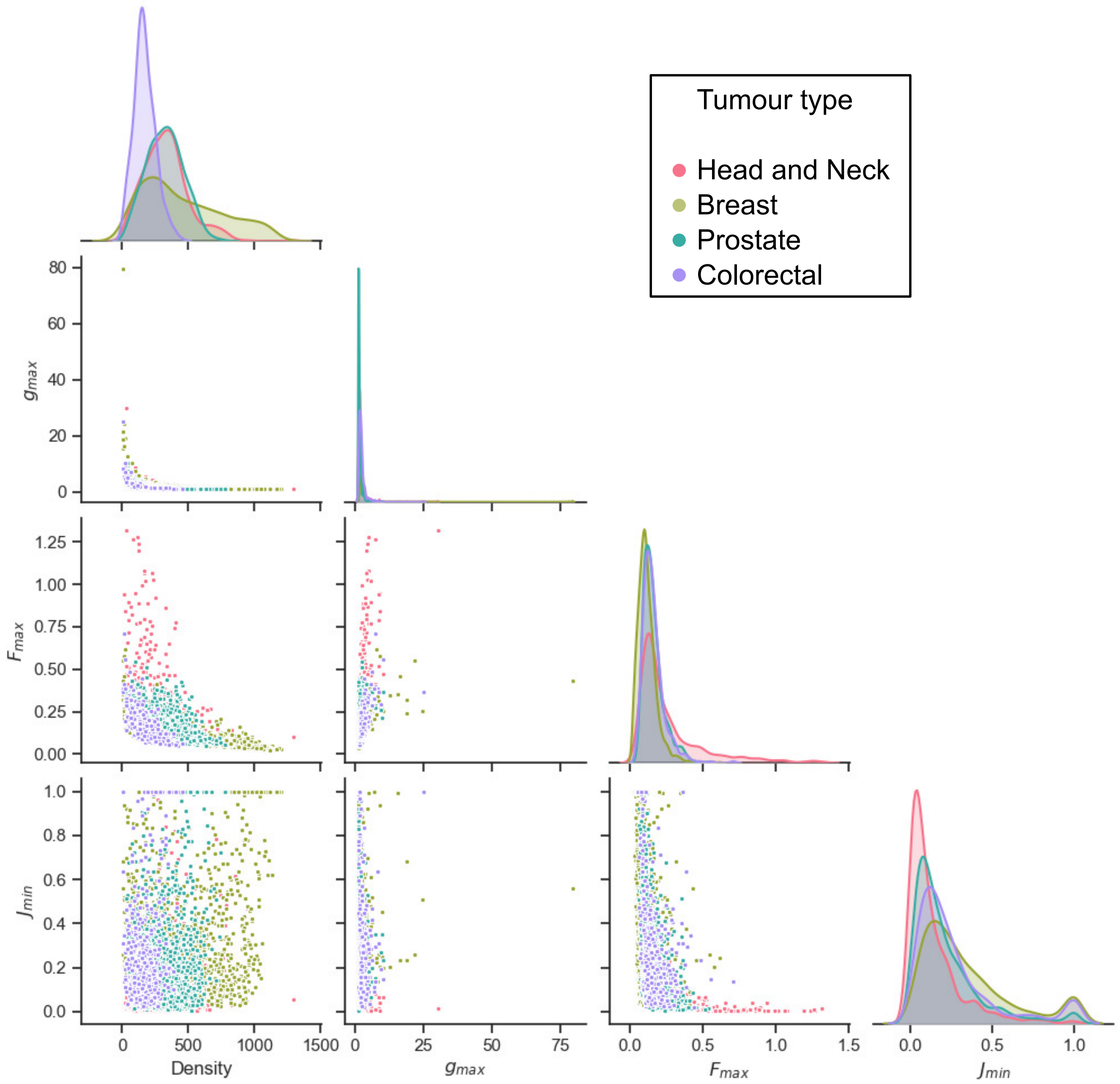

Figure F.1. Distributions (diagonal) and pairwise combinations of density, $g_{\max}$, $F_{\max}$ and $J_{\min}$ for ROIs of varying sizes taken from different tumour indications (549 head and neck, 1.5 mm × 1.5 mm; 1,378 breast, 0.75 mm × 0.75 mm; 429 colorectal, 3 mm × 3 mm; 1,208 prostate, 1.5 mm × 1.5 mm).

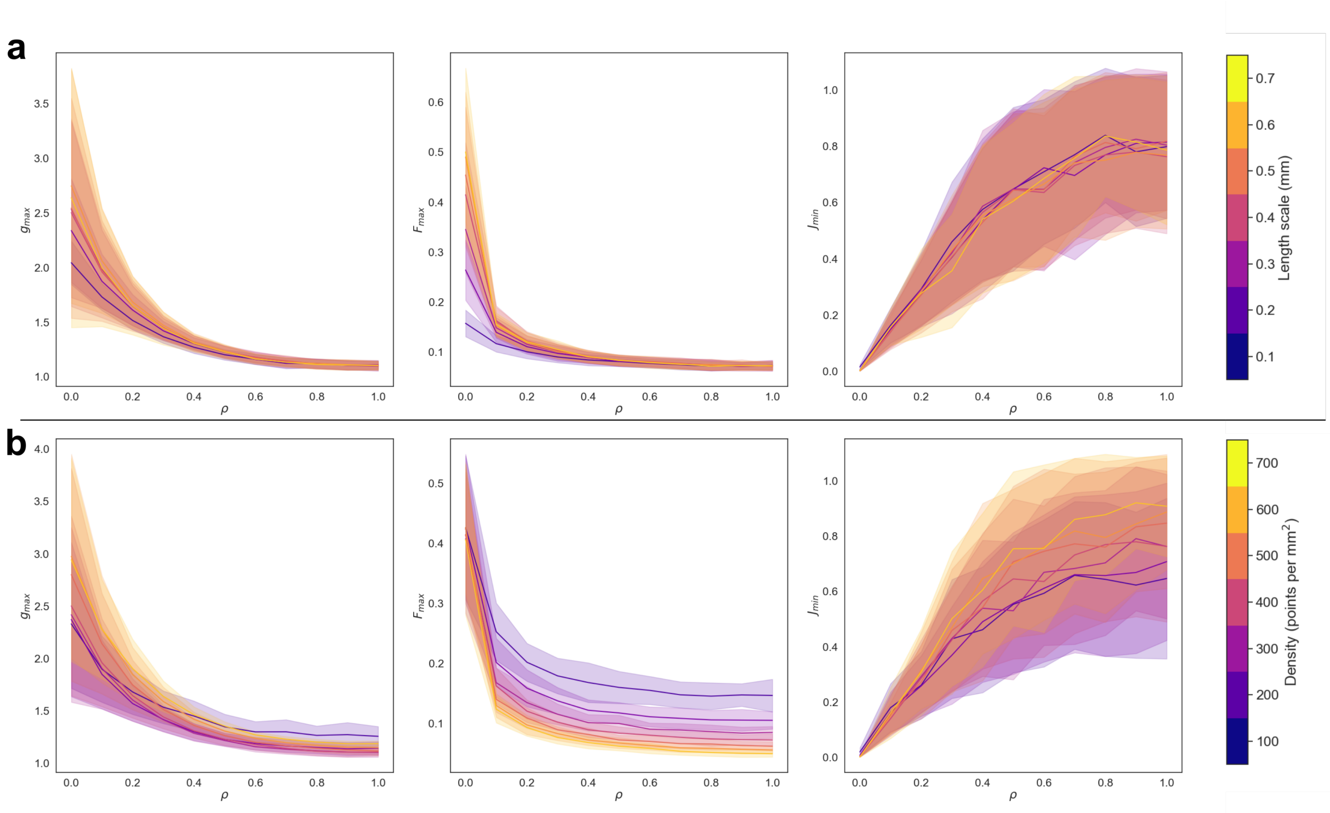

Figure G.1. The effect of varying ρ on $g_{\max}$, $F_{\max}$ and $J_{\min}$ in synthetic data with (a) fixed cell density $d = 400$ cells per mm^2 but varying length scale, and (b) fixed length scale $l = 0.7$ mm but varying cell density. Mean and standard deviation of 100 iterations per ρ for each density and length scale.

observations of $F_{\max}$. This is because when d is high, gaps between points must be narrower than when d is low.

We conclude that although d and l cause some variation in $g_{\max}$, $F_{\max}$ and $J_{\min}$, the main effect of this is to cause a wider range of observed values of $g_{\max}$, $F_{\max}$ and $J_{\min}$ for a given ρ while maintaining the same trend. Since real tumour nests may exhibit a range of length scales and immune cell densities, we incorporate this variation into the training and testing data by choosing d and l randomly for all point patterns with a given ρ. We also note that by definition, ρ is independent from both d and l, since for a given l and d points can be placed with arbitrary ρ.

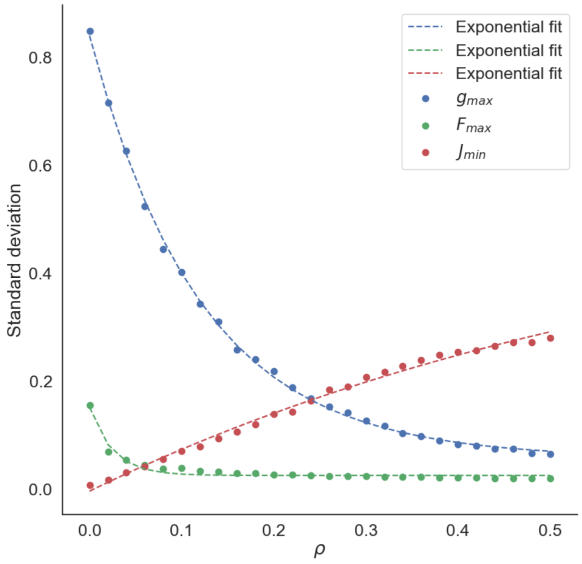

Figure H.1. The standard deviation of the distributions of $g_{\max}$, $F_{\max}$ and $J_{\min}$ for different values of ρ (dots) can be estimated using exponential functions (dashed lines).

H Assumptions in likelihood estimation

H.1 Fitting standard deviations using exponential functions

In Figure 3 we use exponential functions to estimate the means of the distributions of $g_{\max}$, $F_{\max}$ and $J_{\min}$ for different values of ρ:

$$\text{mean}(g_{\max}) = 1.34e^{-4.52\rho} + 1.10 \tag{4}$$

$$\text{mean}(F_{\max}) = 0.18e^{-9.16\rho} + 0.09 \tag{5}$$

$$\text{mean}(J_{\min}) = -2.04e^{-0.74\rho} + 2.06. \tag{6}$$

Figure H.1 shows how the standard deviations of these distributions can also be approximated by exponential functions:

$$\text{SD}(g_{\max}) = 0.78e^{-8.26\rho} + 0.06 \tag{7}$$

$$\text{SD}(F_{\max}) = 0.13e^{-39.54\rho} + 0.03 \tag{8}$$

$$\text{SD}(J_{\min}) = -0.57e^{-1.46\rho} + 0.57. \tag{9}$$

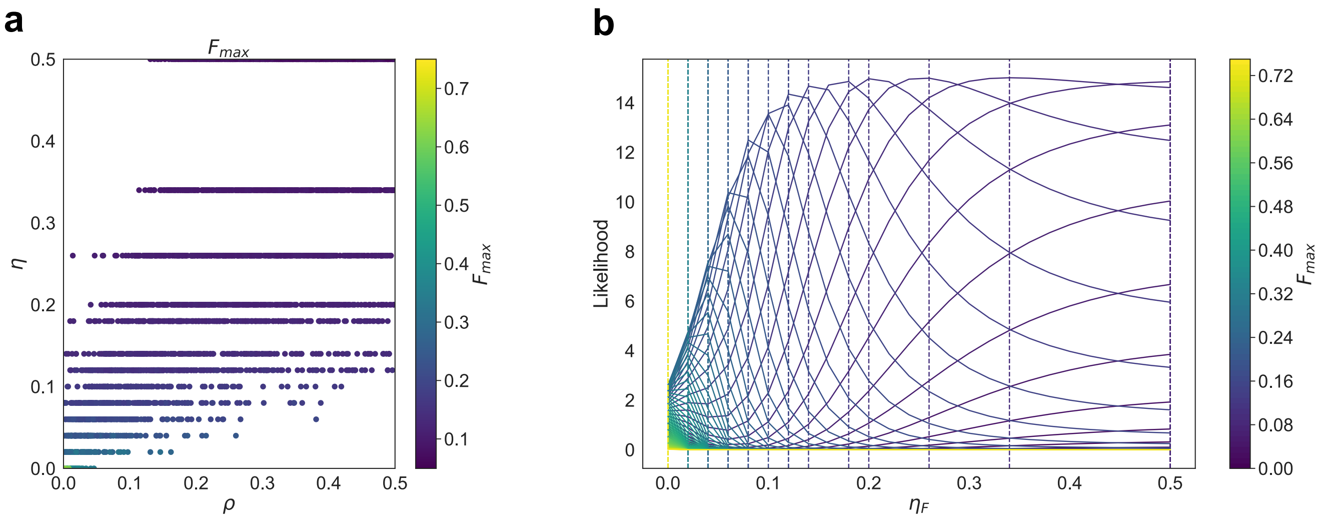

Figure H.2. (a) Reproduction from Figure 4 of ρ vs η_F, now coloured according to $F_{\max}$. (b) Likelihood functions for possible values of η_F for a given observation of $F_{\max}$, with $F_{\max}$ to 2 decimal places. Vertical dashed lines indicate the maxima of the likelihood functions shown. Rounding observations of $F_{\max}$ to discrete values causes jumps in the possible values that can be taken by η_F, as the maximum of the likelihood function can move significantly between two close discrete values of $F_{\max}$.

H.2 Banding in predictions of η

For some combinations of spatial statistics, predictions η appear to show a preference. This banding effect can be seen in Figure 4, particularly for η_F. This behaviour is caused by our use of a lookup table to calculate profile likelihoods. We generate a table in which values of $F_{\max}$ are rounded to 2 decimal places. When calculating η_F, observations of $F_{\max}$ must therefore be rounded to 2 decimal places, and the resulting profile likelihood is used to identify η_F. In Figure H.2(a) we reproduce the η_F values for the synthetic validation dataset shown in Figure 4, coloured according to $F_{\max}$. The values of η_F which appear frequently correspond to the peaks of the profile likelihood functions shown in Figure H.2(b). Each possible profile likelihood corresponds to a discrete value of $F_{\max}$. η_F is the peak of the likelihood function corresponding to the observation of $F_{\max}$. For small $F_{\max}$, the peaks of these likelihood functions can move by up to 0.15 with a change in $F_{\max}$ of just 0.01. This causes the banding effect shown in Figure H.2(a), since any point pattern with the same first two decimal places of $F_{\max}$ will be assigned the same η_F. This effect could be mitigated by taking more decimal places in observations of $F_{\max}$, which would increase the number of likelihood functions considered and the corresponding number of possible η_F values.

H.3 Estimating maximum likelihood directly from empirical distributions

When calculating the profile likelihoods used to estimate η we assume that for point patterns generated using the same ρ, $g_{\max}$, $F_{\max}$ and $J_{\min}$ are normally distributed. We describe in Figure 3 how the mean and standard deviation of these distributions for any ρ can be estimated using an exponential function. One advantage of making this assumption is that it ensures the mean and standard deviation vary monotonically with changes in ρ. This in turn means that the peaks of the profile likelihoods corresponding with different values of $g_{\max}$, $F_{\max}$ and $J_{\min}$ vary monotonically with $g_{\max}$, $F_{\max}$ and $J_{\min}$. This can be seen in Figure H.2(b), where increasing $F_{\max}$ causes η_F to decrease.

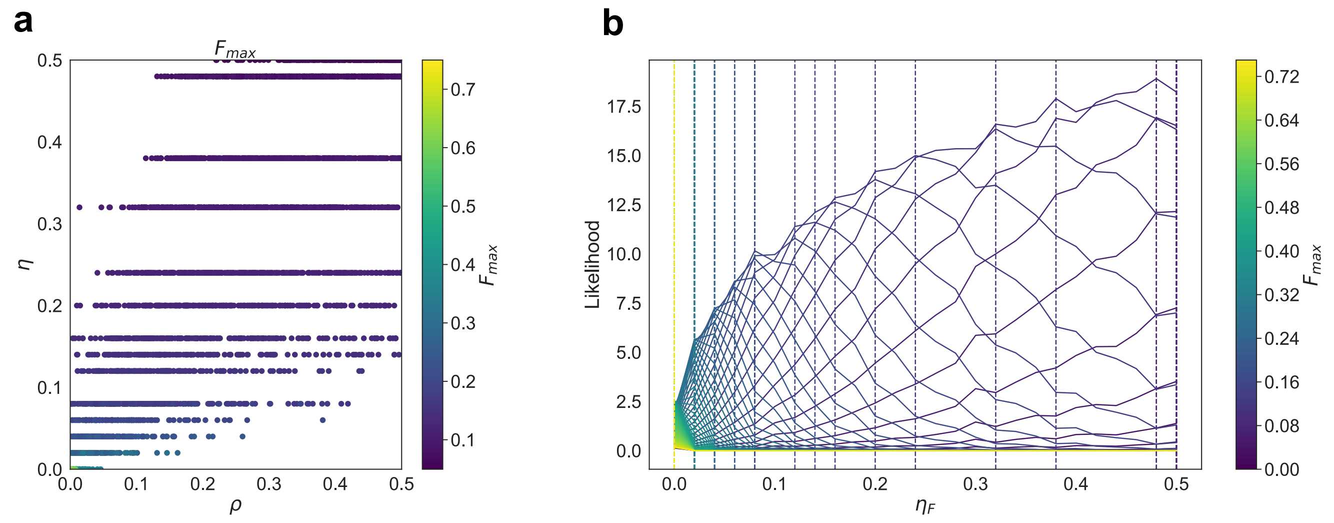

Figure H.3. (a) Predictions of ρ vs η_F for synthetic data coloured according to $F_{\max}$, generated using empirical likelihood distributions. (b) Likelihood functions based on empirical probability distributions from the training data. Vertical dashed lines indicate the maxima of the likelihood functions shown. In contrast to Figure H.2(b), the empirical likelihood functions are not smooth, and their maxima may not vary smoothly with changes in $F_{\max}$.

Instead of estimating the maximum likelihood by assuming that $g_{\max}$, $F_{\max}$ and $J_{\min}$ are normally distributed and then estimating the mean and SD using exponential functions, it is possible to estimate the maximum likelihood directly from the empirical distributions. This results in noisier likelihood functions. Figure H.3(a) shows predictions for η_F for the synthetic testing dataset based on this methodology, with the corresponding likelihood functions and maximum likelihood estimates shown in Figure H.3(b). Using these empirical distributions introduces additional uncertainty into predictions, because the profile likelihoods are not smooth. It also results in profile likelihoods which may have multiple local maxima, which may lead to 95% confidence intervals which contain gaps. These problems could be alleviated by generating more training data, which may cause the empirical likelihoods to become smoother. However, this approach requires referencing the entire training dataset when making maximum likelihood estimations. These disadvantages mean that we instead use the previously described method in which likelihoods are derived from normal distribution approximations to the distributions of $g_{\max}$, $F_{\max}$ and $J_{\min}$.

In Figure H.4 we compare the predictions of η_X obtained using the empirical likelihood distributions (η_X^E) and the continous exponential approximation (η_X^C). Figure H.4 shows that the difference in the predictions is small, suggesting that approximating the empirical distributions with normal distributions whose mean and SD is given by exponential functions of ρ is reasonable.

I Detection of histological artefacts in ROIs using spatial statistics

Several of the 549 head and neck IHC ROIs contain focal areas of missing tissue caused by necrosis, tissue tears and/or holes created on removal of tissue for tissue microarray construction. All missing tissue regions were annotated by a pathologist. These annotated regions do not contain any identifiable macrophages, and therefore appear as voids in point clouds associated with the immune cell locations.

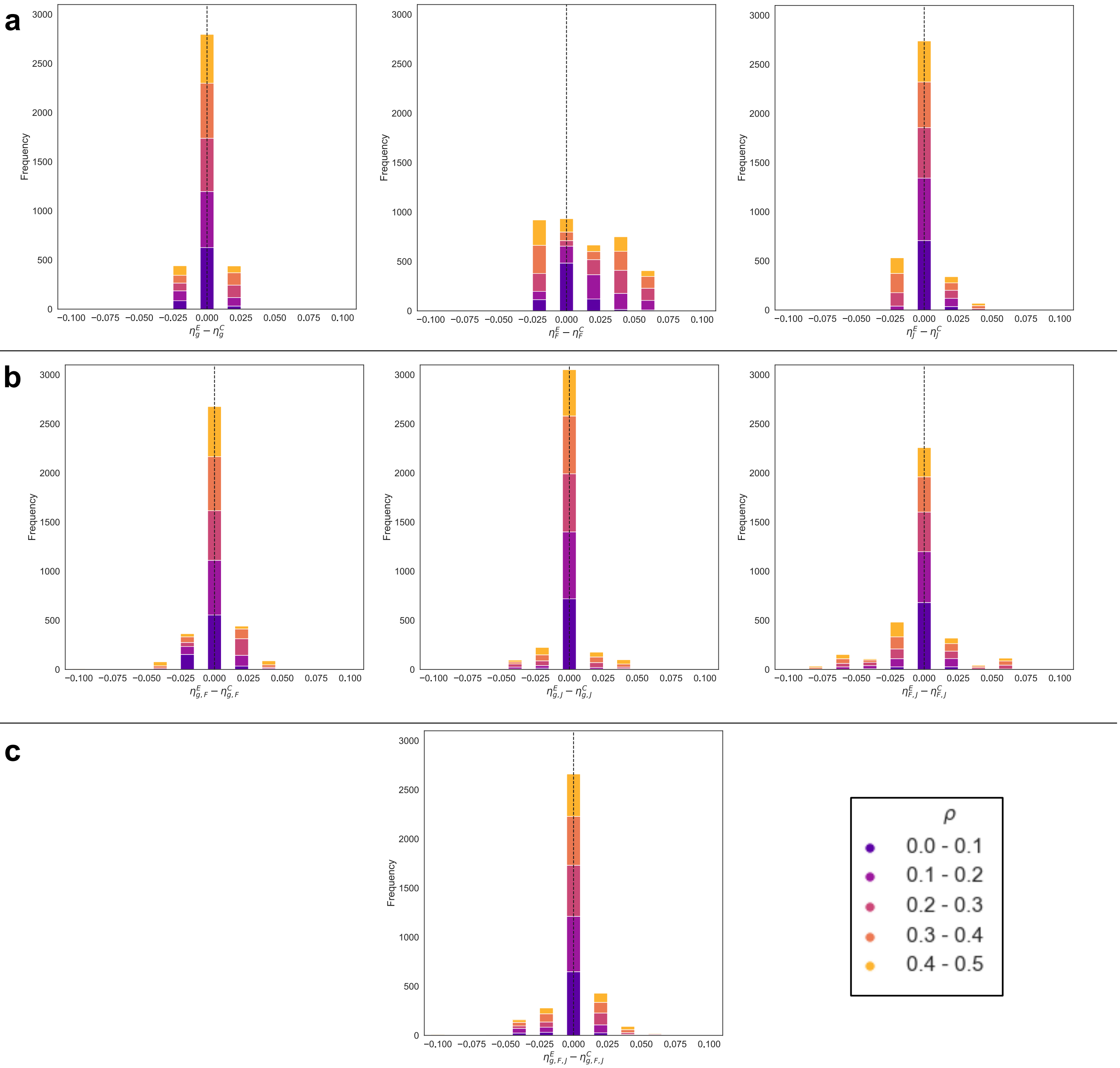

Figure H.4. Difference between η using empirical likelihood distributions (η_X^E) and using the exponential function approximation to the likelihood (η_X^C). For each combination of $g_{\max}$, $F_{\max}$ and $J_{\min}$, the difference between predictions made using the two methods is small, with $\eta_X^E = \eta_X^C$ for most images.

Image	Manual Score	η_g	η_F	η_J	$\eta_{g,F}$	$\eta_{g,J}$	$\eta_{F,J}$	$\eta_{g,F,J}$
(a)	Very Low	0.38	0.26	0.08	0.38	0.34	0.16	0.38
(b)	High	0.24	0.06	0.18	0.08	0.22	0.12	0.12
(c)	High	0.24	0.18	0.06	0.22	0.08	0.06	0.2

Table 3. η values associated with the ROIs in Figure J.1.

Image	Manual Score	Density	$g_{\max}$	$F_{\max}$	$J_{\min}$
(a)	Very Low	683	1.36	0.11	0.15
(b)	High	89	1.64	0.22	0.30
(c)	High	379	1.62	0.13	0.11

Table 4. Density, $g_{\max}$, $F_{\max}$ and $J_{\min}$ values associated with the ROIs in Figure J.1.

Here we show that ROIs containing large areas of necrosis, tissue tears and/or holes, resulting in significant annotation can be identified using spatial statistics. Figure I.1 shows the density of macrophages, $g_{\max}$, $F_{\max}$ and $J_{\min}$ for each of the head and neck ROIs, coloured according to the proportion of the ROI annotated for exclusion. ROIs which have been heavily annotated yield similar spatial statistics, characterised by high values of $g_{\max}$ and $F_{\max}$ and low values of $J_{\min}$. This combination of statistics is the same as that observed in Figure 2 for ROIs with very low levels of macrophage infiltration, or for synthetic data generated using low values of ρ (Figure 3). This suggests that images which are identified as having large immune deserted regions (i.e., very low η) should be checked manually for artefacts.

J ROIs identified as outliers by η

The analysis in Figure 5 contains some ROIs which are consistently misclassified by η. In this Section we present examples of these outliers, and examine why they may be misclassified. Figure J.1 shows three such ROIs, together with the point clouds identified by our image analysis algorithm.

The η values for the ROIs in Figure J.1 are given in Table 3, and the associated spatial statistics are given in Table 4. ROI (a) is often assigned high η, despite being classified as having very low macrophage infiltration into tumour nests. This misclassification is likely due to points being widely spread across the domain and a large amount of stroma in the region. The large number of macrophages identified in the point cloud suggests that the image analysis algorithm used to derive the point cloud also needs refinement in this case, as it appears that dark blue staining has been misidentified as staining for CD68. ROI (b) has high infiltration, but has a very low CD68+ macrophage density of 89 macrophages per mm^2. The statistics we discuss here assign low η to regions with low cell density; this is discussed in detail in Section D of the Supporting Information. ROI (c) has been assessed as having high infiltration, but contains some regions which have no CD68+ cells. In this region the tumour nests are very small relative to the stroma, which makes it difficult for our method to distinguish between tumour cell nests and stroma. Our synthetic data is generated with the assumption that at least 25% of the ROI area is covered by tumour nests to ensure that these areas can be distinguished. It is therefore likely that this ROI was assigned relatively low values of η based on the high local variation of macrophage density within the stroma.

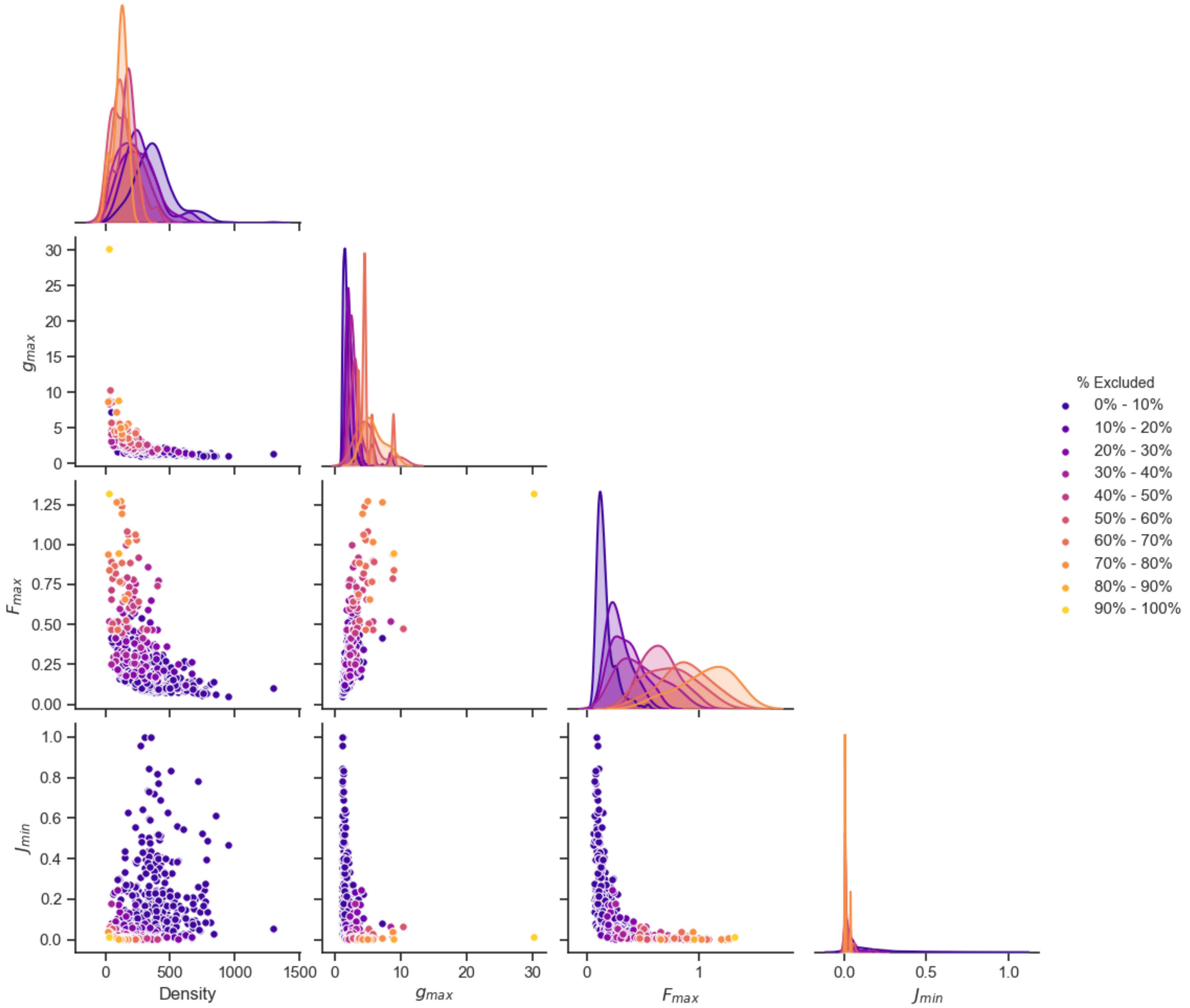

Figure I.1. Distributions (diagonal) and pairwise correlations (off-diagonal) of density, $g_{\max}$, $F_{\max}$ and $J_{\min}$ for the head and neck ROIs, coloured according the proportion of the region with annotations for exclusion. Annotations indicate missing tissue due to necrosis, tissue tears, holes left by removal of cores for tissue microarray construction or other artefacts.

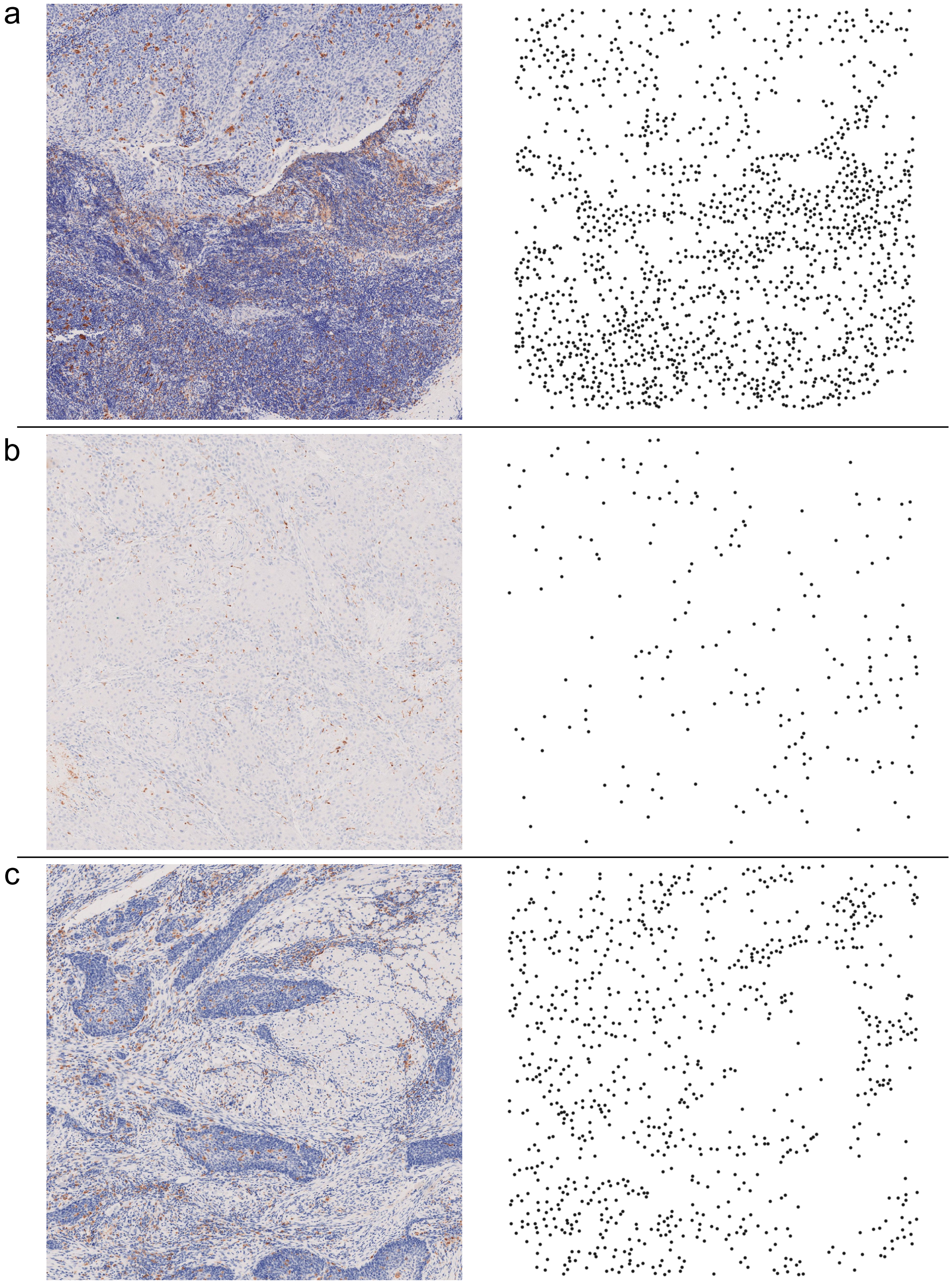

Figure J.1. Three ROIs which are consistently misclassified by η. (a) ROI classified as 'very low' infiltration. (b) ROI classified as 'high' infiltration. (c) ROI classified as 'high' infiltration.

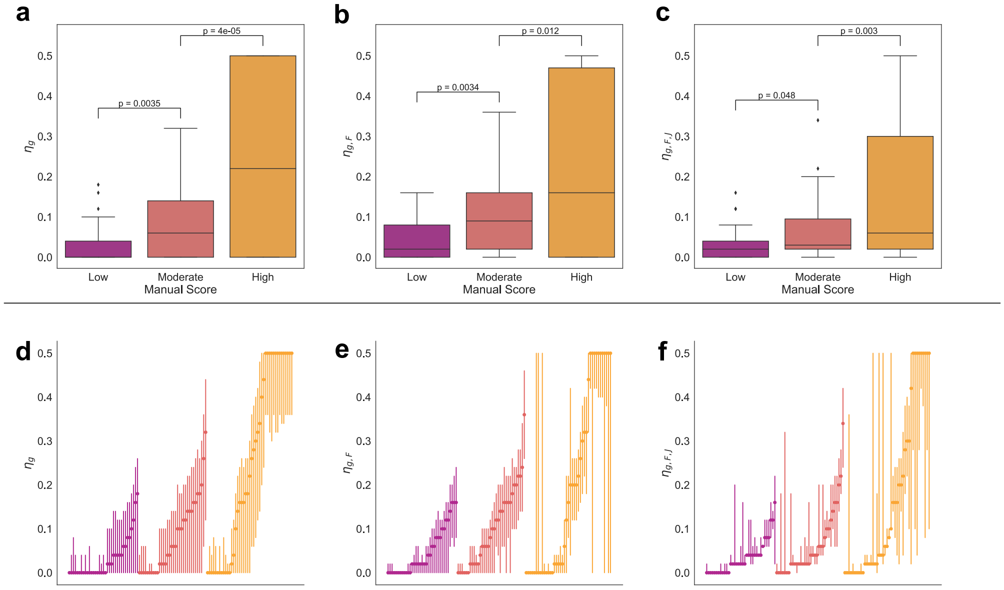

Figure K.1. The effect of including additional observations of spatial statistics on predictions of η applied to point patterns from images showing CD8+ T cell locations within 1.5 mm $\times$ 1.5 mm ROIs. The statistics are predictive of manual classifications by a pathologist. We obtain similar results to Figure 5, which contains a similar analysis of CD68+ macrophage locations. Panels (a)-(c) show η_g, $\eta_{g,F}$ and $\eta_{g,F,J}$ for the 112 ROIs. Panels (d)-(f) show the 95% confidence intervals around η_g, $\eta_{g,F}$ and $\eta_{g,F,J}$ for each ROI, coloured according to the pathologists manual scoring.

K Prediction of η for CD8+ cell infiltration

In the main text, our analysis focussed on quantification of CD68+ infiltration. However, the spatial statistics can be applied to point patterns from different sources. In this section, we apply a similar analysis to CD8+ T cells extracted from IHC images of the same head and neck tumours. As before, 1.5 mm $\times$ 1.5 mm ROIs were extracted from the larger slides. 150 ROIs were randomly selected, and after images with substantial missing tissue or artefacts were excluded, 112 ROIs were classified by a pathologist as containing ‘very low’, ‘low’, ‘moderate’ or ‘high’ CD8+ cell infiltration into tumour nests. No images were assessed as having ‘very low’ infiltration.

Figure K.1 shows how including observations of more spatial statistics affects our ability to infer η for these data (see Figure 5, main text, for similar analysis for CD68+ cells). η_g, $\eta_{g,F}$, and $\eta_{g,F,J}$ can all distinguish regions with low, moderate and high infiltration. As in Figure 5, the error bars become narrower as more spatial statistics are considered.

In common with the analysis of CD68+ macrophages, we note that some ROIs are consistently misidentified (see Section J for a discussion). In particular, we highlight the role that missing tissue plays in misclassification. Most ROIs assigned $\eta = 0$ contain a void in the point pattern, caused by missing tissue or regions of necrosis which are excluded from the analysis.

References

1. Ren, X. & Malik, J. Learning a Classification Model for Segmentation. *Proceedings of the Ninth IEEE International Conference on Computer Vision - Volume 2* **1**, 10—- (2003).

2. Beck, A. H. *et al.* Systematic Analysis of Breast Cancer Morphology Uncovers Stromal Features Associated with Survival. *Science Translational Medicine* **3** (2011).

3. Nguyen, L. *et al.* Spatial Statistics for Segmenting Histological Structures in H&E Stained Tissue Images. *IEEE Transactions on Medical Imaging* **36**, 1522–1532 (2017).

4. Bankhead, P. *et al.* QuPath: Open source software for digital pathology image analysis. *Scientific Reports* **7**, 16878 (2017). URL http://www.nature.com/articles/s41598-017-17204-5.

5. Stutz, D., Hermans, A. & Leibe, B. Superpixels: An evaluation of the state-of-the-art. *Computer Vision and Image Understanding* **166**, 1–27 (2018). URL https://doi.org/10.1016/j.cviu.2017.03.007. 1612.01601.

6. Achanta, R. *et al.* SLIC Superpixels Compared to State-of-the-Art Superpixel Methods. *IEEE Transactions on Pattern Analysis and Machine Intelligence* **34**, 2274–2281 (2012).

7. Cortes, C. & Vapnik, V. Support-Vector Networks. *Machine Learning* **20**, 273–297 (1995). arXiv:1011.1669v3.

8. Lucchi, A., Smith, K., Achanta, R., Lepetit, V. & Fua, P. A Fully Automated Approach to Segmentation of Irregularly Shaped Cellular Structures in EM Images. In Jiang, T., Navab, N., Pluim, J. & Viergever, M. (eds.) *Medical Image Computing and Computer-Assisted Intervention – MICCAI 2010 13th International Conference, Beijing, China, September 20-24, 2010, Proceedings, Part II*, 463–471 (Beijing, China, 2010).

9. Lucchi, A. *et al.* Learning structured models for segmentation of 2-D and 3-D imagery. *IEEE Transactions on Medical Imaging* **34**, 1096–1110 (2015).

10. El-Naqa, I., Yang, Y., Wernick, M. N., Galatsanos, N. P. & Nishikawa, R. M. A support vector machine approach for detection of microcalcifications. *IEEE Transactions on Medical Imaging* **21**, 1552–1563 (2002).

11. Chaplot, S., Patnaik, L. M. & Jagannathan, N. R. Classification of magnetic resonance brain images using wavelets as input to support vector machine and neural network. *Biomedical Signal Processing and Control* **1**, 86–92 (2006).

12. Arteta, C., Lempitsky, V., Noble, J. A. & Zisserman, A. Learning to Detect Cells Using Non-overlapping Extremal Regions. In *Medical Image Computing and Computer-Assisted Intervention – MICCAI 2012*, 348–356 (2012). URL http://link.springer.com/10.1007/978-3-642-33415-3{_}43. 9780201398298.

13. Romero Castro, E. *et al.* A watershed and feature-based approach for automated detection of lymphocytes on lung cancer images. *Medical Imaging 2018: Digital Pathology* 26 (2018). URL https://www.spiedigitallibrary.org/conference-proceedings-of-spie/10581/2293147/

A-watershed-and-feature-based-approach-for-automated-detection-of/10.1117/12.2293147.full.

14. Xing, F. & Yang, L. Robust nucleus/cell detection and segmentation in digital pathology and microscopy images: A comprehensive review. *IEEE Reviews in Biomedical Engineering* **9**, 234–263 (2016). 15334406.

15. Chourasiya, S. & Rani, G. U. Automatic Red Blood Cell Counting using Watershed Segmentation. *International Journal of Computer Science and Information Technologies* **5**, 4834–4838 (2014).

16. Veta, M. *et al.* Automatic Nuclei Segmentation in H&E Stained Breast Cancer Histopathology Images. *PLoS ONE* **8**, 1–12 (2013).

17. Schneider, C. A., Rasband, W. S. & Eliceiri, K. W. NIH Image to ImageJ: 25 years of image analysis. *Nature Methods* **9**, 671–675 (2012).

18. Abràmoff, M. D., Magalhães, P. J. & Ram, S. J. Image processing with imageJ. *Biophotonics International* **11**, 36–41 (2004).

19. Johnston, S. T. & Crampin, E. J. Corrected pair correlation functions for environments with obstacles. *Physical Review E* **99**, 1–19 (2019).

20. Adler, R. J. *The geometry of random fields* (Society for Industrial and Applied Mathematics, 2010).

21. Błaszczyszyn, B. & Yogeshwaran, D. Clustering Comparison of Point Processes, with Applications to Random Geometric Models. In Schmidt, V. (ed.) *Stochastic Geometry, Spatial Statistics and Random Fields: Models and Algorithms*, chap. 2, 31–71 (Springer, 2015), electronic edn.

22. Hessel, H. *et al.* Tumor associated macrophages in breast cancer and their potential role in tumor prognosis. In *98. Jahrestagung der Deutschen Gesellschaft für Pathologie e.V.* (Berlin, 2014).
